# Symbiont Diversity of Rice-Associated Leafhoppers (Cicadellidae) in the Tropical Floodplains of the Tonle Sap Lake, Cambodia

**DOI:** 10.1007/s00248-025-02619-9

**Published:** 2025-10-17

**Authors:** Sophany Phauk, Lorenzo Assentato, Sopha Sin, Onnorong Uk, Sophorn Hap, Olle Terenius

**Affiliations:** 1 Department of Biology, Faculty of Science, Royal University of Phnom Penh, Phnom Penh, Cambodia; 2 Department of Cell and Molecular Biology, Microbiology, Uppsala University, Uppsala, Sweden; 3 Centre for Biodiversity Conservation, Faculty of Science, Royal University of Phnom Penh, Phnom Penh, Cambodia

**Keywords:** Leafhoppers, Cicadellidae, Symbiont, Bacterial community, 16S rRNA, Tonle Sap Lake

## Abstract

Rice-associated leafhoppers (Cicadellidae) play a significant role in rice agroecosystems, contributing not only to direct crop damage but also to the transmission of plant pathogens. This study investigates the symbiont diversity of seventeen leafhopper species from the tropical floodplains of Tonle Sap Lake (TSL), Cambodia. The dominant symbiont across most species was *Candidatus (Ca.)* Karelsulcia muelleri, an obligate primary endosymbiont essential for nutrient synthesis. The co-obligate symbiont *Ca.* Nasuia deltocephalinicola was also consistently detected, particularly in Deltocephalinae hosts. In addition, several secondary symbionts, including *Sodalis, Arsenophonus, Diplorickettsia, Rickettsia, Wolbachia*, and *Ca.* Lariskella, were identified, showing species-specific associations and potential roles in host fitness and pathogen transmission. Variations in symbiont diversity were observed across cicadellid species, geographic origins, and between sex-associated symbionts, with notable differences in the bacterial composition of *Nephotettix virescens*. While geographical differences (Battambang vs. Kampong Thom) did not strongly affect microbial composition, sex-associated variations were evident in *N. virescens*. Females exhibited a higher abundance of *Karelsulcia* and *Nasuia*, suggesting possible microbial adaptation related to reproduction. This study highlights the complex and dynamic nature of cicadellid hosts–symbiont interactions and suggests that microbial communities are primarily structured by host species. While geographic distance can influence these communities, this effect is not the same for every species. These findings provide critical insights into the microbial diversity of rice-associated leafhoppers and their potential for ecological roles in rice farming systems. Further studies, including functional analysis and host-symbiont interactions, are crucial to understanding the ecological roles and evolutionary dynamics of these microbial communities.

**Supplementary Information:**

The online version contains supplementary material available at 10.1007/s00248-025-02619-9.

## Introduction

Rice-associated leafhoppers (Auchenorrhyncha: Cicadellidae) are a diverse group of sap-feeding insects widely associated with a variety of agricultural ecosystems worldwide [1]. In Southeast Asia, where rice agriculture is a vital component of global food security, millions rely on rice cultivation for sustenance and economic stability [2]. However, rice crop ecosystems are highly susceptible and prominent to leafhoppers. These insects feed on plant sap and are commonly observed across rice-growing regions, often in high densities [1, 3, 4]. Several species, including *Nephotettix virescens*, *Cofana spectra*, and *Exitianus indicus*, are frequently found in the paddy rice fields and have developed strong associations with rice plants throughout their life cycles [5]. Their prevalence and biological interaction with host plants give them important ecological roles in paddy habitats.

The family *Cicadellidae* includes a diverse array of species with varying ecological roles, ranging from generalist herbivores to major agricultural pests [6]. In rice ecosystems, cicadellid leafhoppers are of particular concern, as they not only cause direct damage by feeding on plant sap, but also serve as significant vectors of plant pathogens, including rice tungro virus [1, 5, 6] and phytoplasmas [7]. For instance, species of *Nephotettix* are known to transmit these agents, resulting in substantial economic losses in rice production [1]. Although insect feeding alone can weaken plant vigor, the primary threat arises from their role in spreading diseases across large agricultural areas [1, 5, 8]. These combined impacts make cicadellids major drivers of economically significant disease outbreaks across Southeast Asia [1, 3, 6].

The ecological diversity and adaptability of rice-associated leafhoppers to different environmental conditions make them important subjects for both agricultural and ecological research [3]. Effective pest management strategies require a comprehensive understanding of their species composition, population dynamics, interactions with host plants and microorganisms, natural enemies, and environmental factors [1, 3, 4, 8]. This need is particularly important in floodplain regions such as those surrounding the Tonle Sap Lake (hereafter TSL), where rice cultivation is especially vulnerable to pest pressures [1]. For instance, *N. virescens* has been consistently reported as the dominant species in paddy fields in Nepal [4]. These insects thrive in the floodplain ecosystem surrounding TSL, where environmental variations and diverse plant hosts shape their population structures. Given their local abundance and ecological importance as herbivores and pathogen vectors in rice fields [1, 3, 5, 6], studying the composition and interactions of cicadellid species in this dynamic agricultural landscape is essential.

Microorganisms are incredibly diverse and thrive in nearly every environment on Earth; however, the microbiota associated with eukaryotic organisms have garnered significant scientific interest due to their ecological and agricultural importance [9]. Host-microbe interactions vary widely in both taxonomy and function, especially in arthropods, ranging from mutualism and commensalism to pathogenicity. These relationships often have profound effects on host biology and evolution [10]. In insects, symbionts are generally classified into three major categories. First, obligate nutritional endosymbionts are intracellular microbes that are maternally inherited and essential to host survival, development, and reproduction. They compensate for dietary deficiencies by supplying vital nutrients such as amino acids and vitamins [11, 12]. The second group, facultative endosymbionts, varies in prevalence among species, populations, and tissues, and are not required for host survival. Nonetheless, they often confer adaptive advantages, such as enhancing resistance to environmental stress or manipulating host reproductive systems [12]. Lastly, a wide range of microbes colonize the insect gut or external body surfaces. Some of these microbes form long-standing, co-evolved associations with their hosts, while others establish more transient or opportunistic interactions [12].

Rice-associated leafhoppers (Cicadellidae) maintain complex relationships with microbial symbionts that influence their survival, reproduction, and adaptation to environmental pressures [13–17]. Obligate symbionts such as *Candidatus (Ca)* Karelsulcia muelleri (hereafter *Karelsulcia*) and their co-obligate *Ca.* Nasuia deltocephalinicola (hereafter *Nasuia*) [18] are essential for supplementing the leafhopper’s diet with nutrients missing from phloem sap [16, 19]. Meanwhile, secondary (facultative) symbionts, including *Wolbachia, Arsenophonus*, and *Rickettsia*, can affect host biology in diverse ways, including altering reproduction, host fitness, pathogen transmission, and potential insecticide resistance [9, 20]. One example is the protective effects of *Arsenophonus* and *Wolbachia*, either individually or in co-infection, in the planthopper *Nilaparvata lugens* against chemical insecticides [21]. Therefore, investigating the symbiotic community structure associated with cicadellid hosts in the rice field habitats of the tropical floodplains in the TSL region may provide valuable insights into their ecological roles.

Despite their importance, relatively little is known about how symbiotic communities vary among rice-associated leafhopper species or how they are influenced by host-related environmental factors. To address this gap, our study was designed with three specific objectives: (i) to characterize interspecific variation in symbiotic communities among rice-associated leafhopper species, (ii) to evaluate how geographic location (Battambang vs. Kampong Thom provinces), which differ in ecological conditions, shapes microbial assemblages within the TLS floodplain region, and (iii) to investigate sex-associated differences in the microbial composition of the major pest species *Nephotettix virescens*. Based on previous studies of Auchunorrhyncha symbiosis [13, 16, 19], we hypothesized that obligate symbionts (e.g., *Karelsulcia*) would be highly conserved within host lineages, whereas secondary symbionts would vary according to host species, geographic origin, seasonal factors, and sex. By addressing these aspects, this study provides new insights into the community dynamics of host-symbiont interactions in rice-associated leafhoppers, contributing essential knowledge to inform pest management strategies in vulnerable rice-farming systems.

## Materials and Methods

### Study Site

**Fig. 1:**
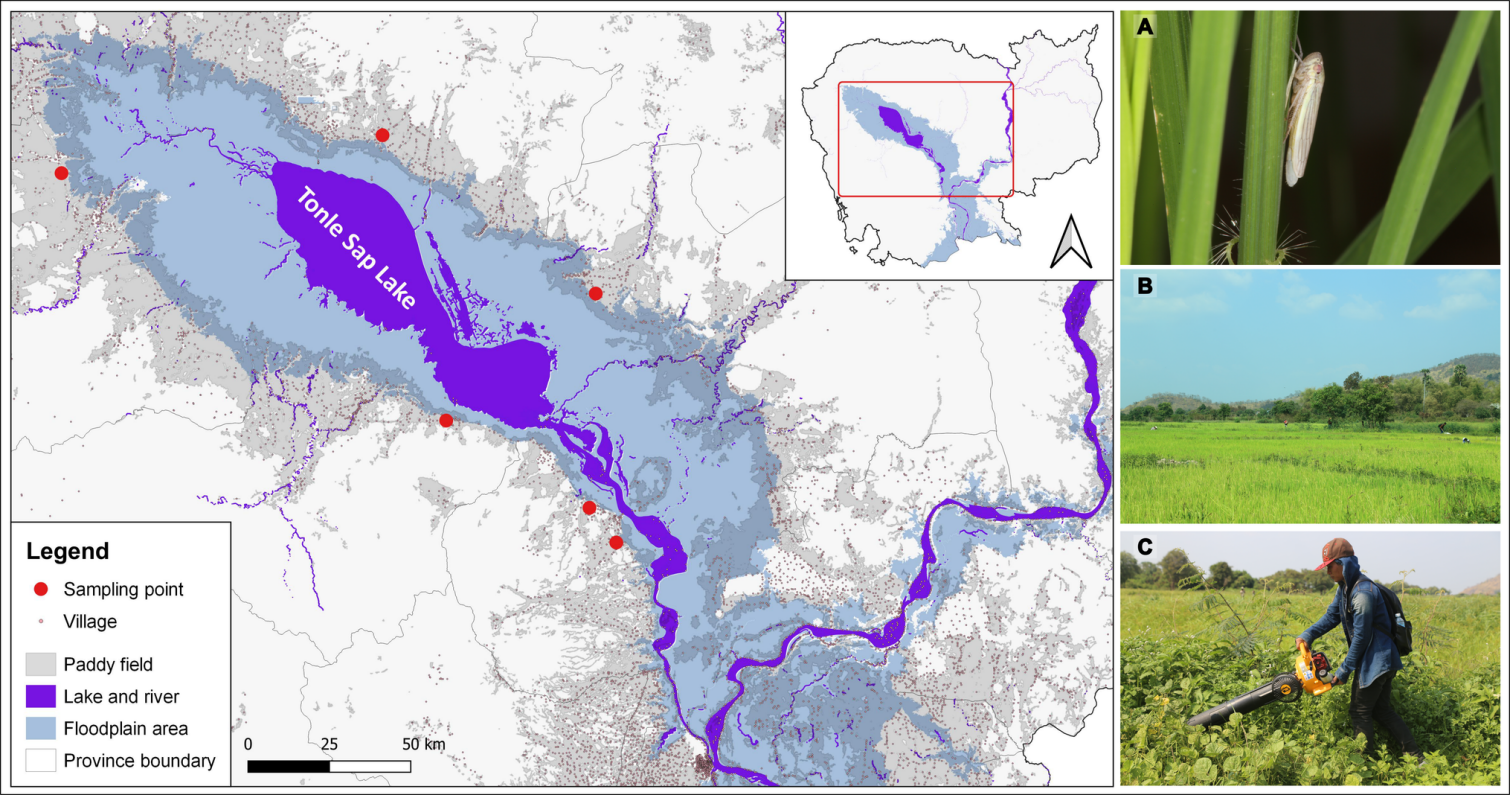
Geographical distribution of sampling sites along the floodplain habitats of the Tonle Sap Lake (TSL). **A** White leafhopper (*Cofana spectra*) feeding on the sap of rice plants. **B** An overview of rice crop habitat, one of the sampling sites in the Kampong Chhnang province. **C** D-Vac vacuum sampler, one of the methods used for insect collection

The Tonle Sap biosphere reserve, including the Tonle Sap Lake in Cambodia, the largest freshwater lake in Southeast Asia, supports extensive rice cultivation along its floodplains [22]. The lake is geographically located in the Northwest of Cambodia and is bordered by several key rice-production provinces (Siem Reap, Battambang, Kampong Thom, Pursat, and Kampong Chhnang). The region’s unique hydrological cycle, characterized by seasonal flooding and receding waters, influences rice production and pest population dynamics [23]. Research sampling was conducted along the Tonle Sap Lake (TSL) [12° 55′ 39.50″N, 104° 3′ 13.22″E; Evl. 4 m], Cambodia (Fig. 1; Table S1). TSL is a major natural reservoir connected to the Mekong River and is the largest natural inland lake in Southeast Asia [24]. From May to October, water flows from the river into TSL, whereas from November to April, water flows out of the lake. The seasonal waterflow (inflow and outflow) expands the nutrition and benefits for aquatic biodiversity and agricultural areas along the TSL [22].

#### Sample Collection

Cicadellid sampling was conducted by using a sweep-net and a D-Vac vacuum sampler (Fig. 1C). Insect samples were collected at four different timepoints in November 2019 and in January, July, and September 2020. Aspirators were used to transfer selected collected Cicadellids into Eppendorf 1.5 mL vials and preserved in 95% ethanol. All collected materials were brought to the Cambodian Entomology Initiatives (CEI) at the Royal University of Phnom Penh in Cambodia for sorting and preliminary identification into morphospecies.

Seventeen rice-associated hopper species were selected for this study, belonging to three subfamilies: Cicadellinae (*Cofana spectra*)*,* Iassinae (*Batracomorphus angustatus*), and Deltocephalinae (*Exitianus indicus, Nephotettix nigropictus, Nephotettix virescens, Maiestas dorsalis, Maiestas* sp., *Goniagnathus punctifer, Hecalus arcuatus, Hecalus* sp.*, Balclutha* sp.*, Hishimonus* sp.*, Neodartus* sp.*, Stirellus capitatus, Stirellus* sp1.*, Stirellus* sp2., and unidentified species belonging to *Deltocephalini* sp.). Morphological identification of 17 leafhopper species was performed by the first author and subsequently confirmed by Christopher H. Dietrich at Illinois Natural History Survey (INHS). To support morphological identification, molecular confirmation was conducted by amplifying the mitochondrial cytochrome c oxidase subunit I (COI) gene using universal primers (LCO1490/HCO2198) for some selected samples. BLAST searches against the NCBI and BOLD databases generally confirmed identifications at the genus level, which was consistent with our morphological assignments. The resulting COI sequences were subsequently used to construct the host phylogenetic tree presented in Fig. 4 (Supplementary Figure S1).

#### DNA Extraction

A total of 126 cicadellid samples (whole tissues) were DNA extracted and purified by using the QIAamp DNA Mini Kit Protocol (Qiagen) with the addition of 20 mg mL^−1^ of lysozyme enzyme (Table S1). The final elution DNA template of 125 µL per sample was used in the study. To ensure data quality, two templates of microbial community standards (*Zymo*BIOMICs, Zymo Research, Irvine, CA, USA) were included as positive controls (Figure S2).

### PCR Amplification and High-Throughput Sequencing

PCR amplification of extracted DNA (*n* = 126) was performed by using a two-step method as described in [25] to generate barcoded bacterial 16S rDNA gene amplification for sequencing. The bacterial 16S rDNA target regions of interest (V3–V4) were amplified by using general bacterial primers 341 F (5′-CCT ACG GGN GGC WGC AG-3′) and 805R (5′-GAC TAC HVG GGT ATC TAA TCC-3′) [26]. Per PCR reaction, 60–100 ng DNA was used as templates in the first step PCR. The first-step PCR program was performed by an initial denaturation at 95 °C for 5 min, followed by 25 cycles of [95 °C for 40 s, 53 °C for 40 s, and 72 °C for 60 s], and final elongation at 72 °C for 7 min. First step-PCRs were analyzed by using Gel Electrophoresis and PCR products were quantified by Image Lab 6.0 software. Sample templates were all applied 25 cycles for the first step-PCR. PCR products were diluted in nuclease-free water to a concentration of 0.1–1 ng μL^−1^ for the next step [27]. In the second step PCR, 1 µL was used as a template from diluted PCR products and PCR was performed by adding 1 of 50 different barcoding primer pairs; to be able to pool 50 samples per sequencing pool, different barcoding primer pairs were applied (Table S8), following the methods described in Sinclair et al. [28]. The second-step PCR is using the same program as that of the first step-PCR, but only for 10 cycles. The resulting PCR products were pooled, purified, and eluted in 50 µL nuclease-free water [29]. For all PCR reactions, Illustra PuReTaq-To-Go PCR Beads (GE Health Care) were used.

Amplicon sequencing using *MiSeq* technology was carried out at the SNP&SEQ Technology Platform, Science for Life Laboratory (SciLifeLab) at Uppsala University, Sweden (https://snpseq.medsci.uu.se/). Libraries were prepared with 5 ng of DNA per sample. Paired-end sequencing was conducted using 300 bp read lengths and v3 chemistry on the *MiSeq* system (Illumina), according to the manufacturer’s guidelines. To improve sequence quality, a PhiX phage library was included as a 10% spike-in during the sequencing run.

### Bioinformatics

Raw*.fastq* files obtained from the sequencing facility were processed at the Uppsala Multidisciplinary Center for Advanced Computational Science (UPPMAX), under project NAISS 2025/22–339, supported by the Swedish National Infrastructure for Computing (SNIC). Demultiplexing was performed using *Cutadapt* (v. 4.1) [30], targeting paired barcodes (--*pair-adapters*) under default settings. PCR primers (341F-805R) were trimmed, allowing up to 10% mismatches (1 error for the 17-nt forward primer and 2 errors for the 21-nt reverse primer). Reads without identifiable primers were discarded.

Subsequent quality-based trimming was carried out with *TrimGalore* (v. 0.6.7) [31] using default settings. The trimmed reads were processed with *DADA2* (v. 1.26.0) in R (v. 4.2.0) [32], which included error modeling, inference of amplicon sequence variants (ASVs) using pooled processing, and paired-end read merging. ASVs were filtered to retain only sequences of 350–470 nt in length, and chimeras were removed using *DADA2*’s *‘removeBimeraDenovo’* function. Taxonomic classification of ASVs was performed using the *DECIPHER* package (v. 2.26.0) with the *IDTAXA* algorithm [33], aligning both strands against a modified *SILVA SSL* (r138) database [34].

The ASV count table, taxonomy table, and ASV sequence list were generated. The ASV sequences were aligned with *MAFFT* (v. 7.508) [35] using default ‘*-auto*’ settings. This alignment was then used to construct an unrooted phylogenetic tree with *IQ-TREE* (v. 2.2.0.3) [36] employing ModelFinder Pro ‘*MFP*’ for model selection. All data—including the ASV table, taxonomy assignments, and phylogenetic tree—were imported into the *phyloseq* package (v. 1.42.0) [37] for downstream analysis. Within *phyloseq*, ASVs unclassified at the phylum level, or those assigned to Chloroplast or Mitochondria, were filtered out, and the results were assembled in a Phyloseq object. All results were saved as an*.RData* object for further analysis.

### Microbial Analysis and Visualization

**Fig. 2:**
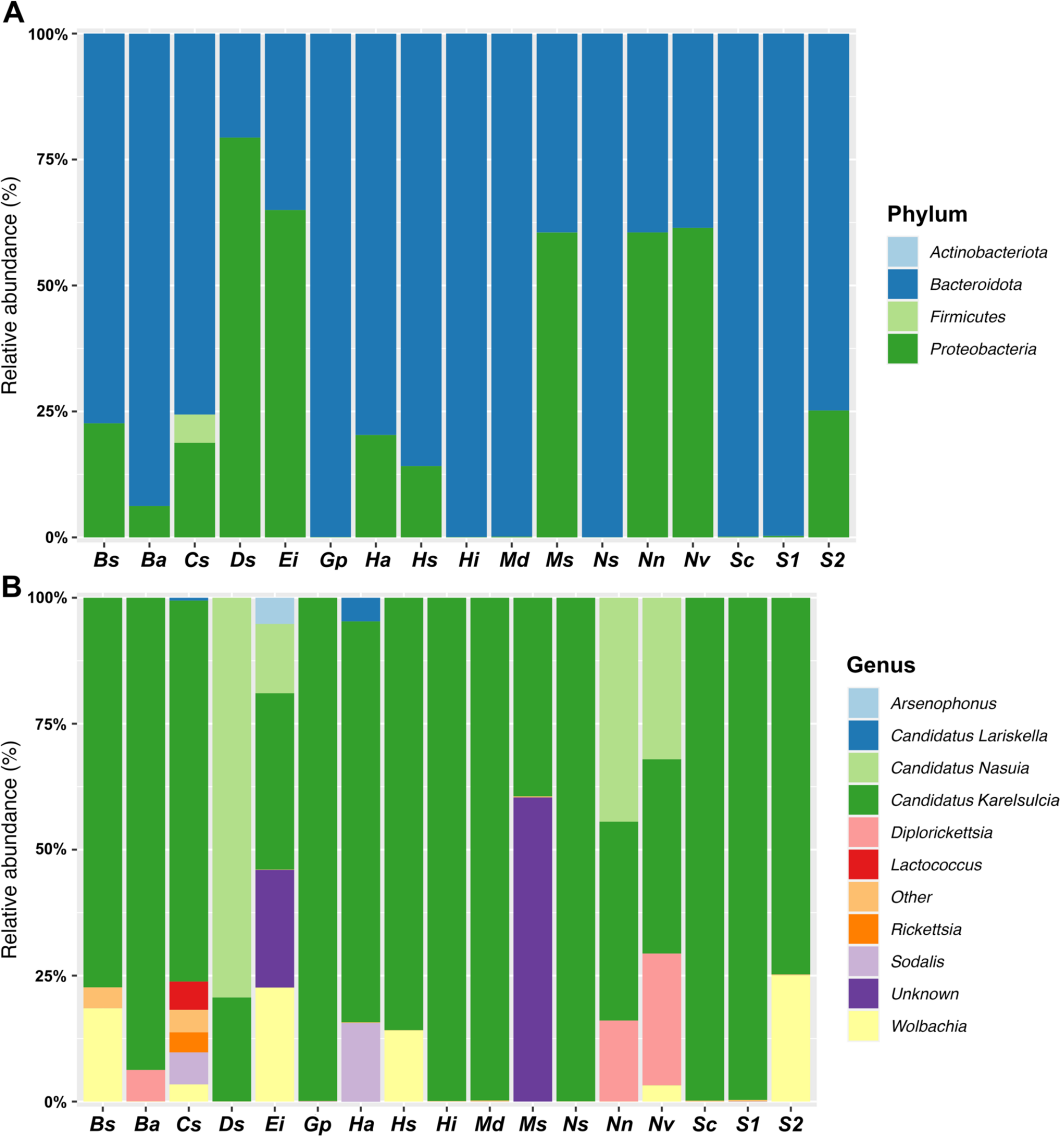
Bacterial composition associated with seventeen leafhoppers (Cicadellidae) species at the Phylum (**A**) and Genus (**B**) levels. The bar plots represent the bacterial composition of leafhopper samples (per species). The abbreviations on the *x-axis* correspond to the following host species: *Balclutha* sp. (Bs)*, Batracomorphus angustatus* (Ba), *Cofana spectra* (Cs)*, Deltocephalini* sp. (Ds), *Exitianus indicus* (Ei)*, Goniagnathus punctifer* (Gp)*, Hecalus arcuatus* (Ha)*, Hecalus* sp. (Hs)*, Hishimonus* sp. (Hi)*, Maiestas dorsalis* (Md)*, Maiestas* sp. (Ms), *Neodartus* sp. (Ns)*, Nephotettix nigropictus* (Nn)*, Nephotettix virescens* (Nv)*, Stirellus capitatus* (Sc)*, Stirellus* sp1. (S1)*,* and *Stirellus* sp2. (S2)

**Fig. 3:**
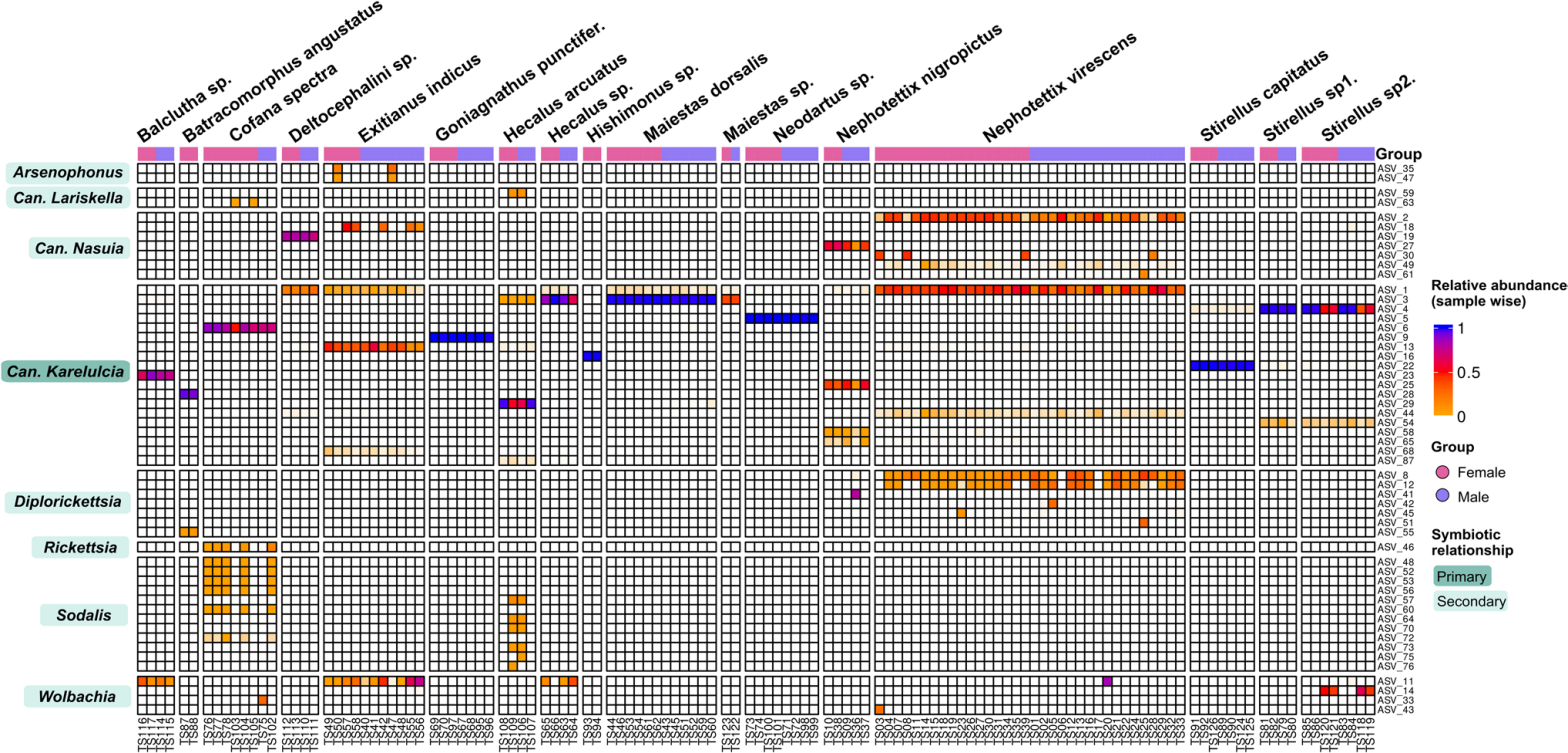
Symbionts associated with *Cicadellidae* hosts from the Tonle Sap Lake: The heatmap shows ASVs of primary/secondary symbionts, and their relative abundance (calculated sample-wise on the total number of reads per sample). To avoid the presence of singletons and low-abundance ASVs the lower limit of the scale has been set to 0.01 (1%) of relative abundance

**Fig. 4:**
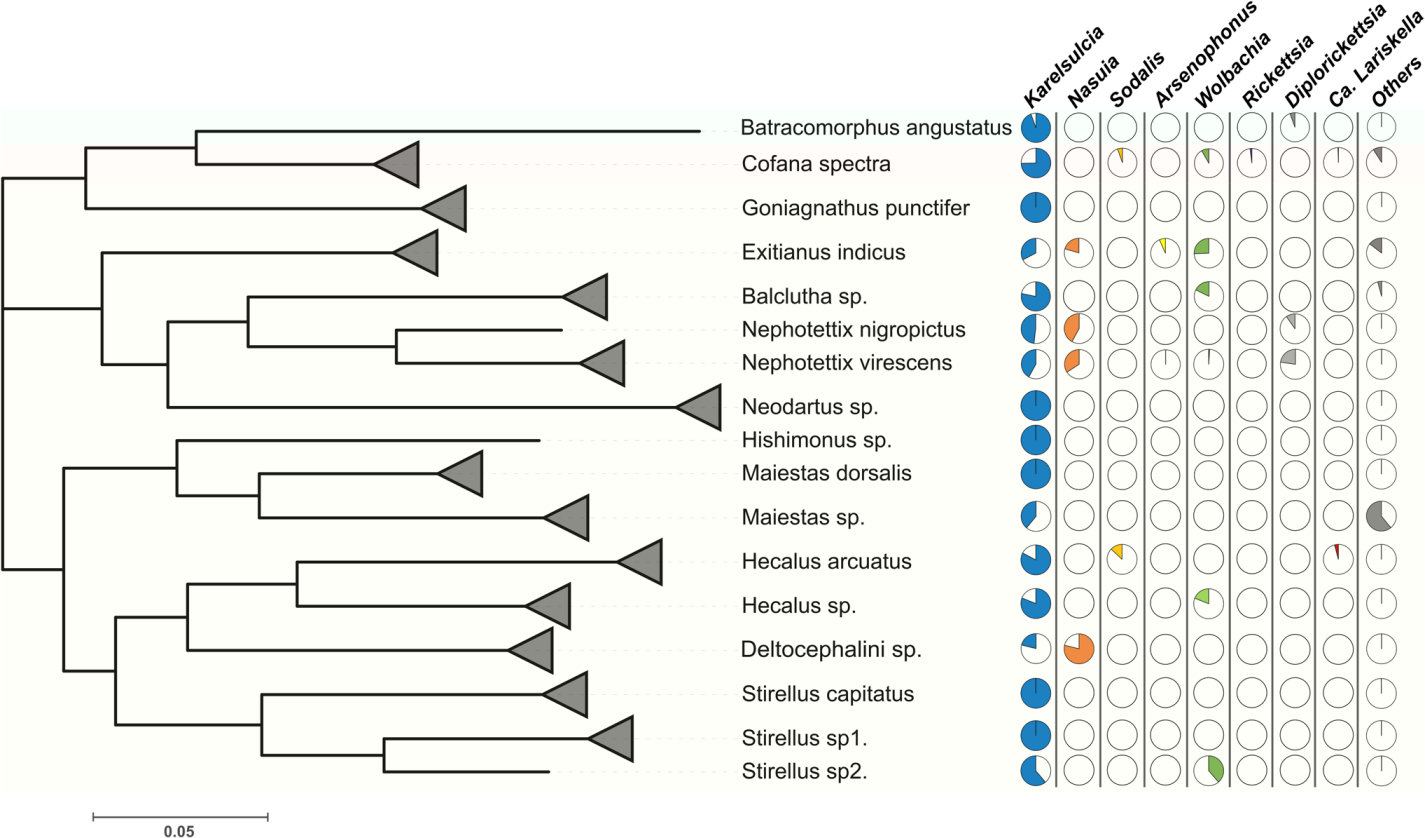
Cicadellidae host-symbiont associations: the graph illustrates the phylogenetic relationships of leafhopper (Cicadellidae) species and their associated symbiotic bacterial communities. On the left, a phylogenetic tree of leafhopper species is shown, based on the COI DNA barcode gene region. The tree was constructed using the Maximum Likelihood (LM) method with bootstrap values set at 1000. Black triangles indicate cases when two or three samples of the sample species were collapsed, as shown in Figure S1. On the right, multiple pie-charts display the relative abundance of symbiotic bacteria associated with each host species

All downstream analyses were conducted in R (v. 4.3.2). Amplicon sequence data were imported and processed into a *phyloseq* object using the *phyloseq* package (v. 1.4.2) [37] for integrated microbiome analysis. To characterize microbial variation across leafhopper hosts, bacterial communities were analyzed at phylum and genus levels. Seventeen rice-associated leafhopper species were analyzed at the level of individual insect samples, treating each specimen as an independent unit. The approach allows for assessing intra- and interspecific microbial variation and is commonly used in host-microbiome studies involving symbiont diversity [9, 38]. Taxonomic composition was visualized using stacked bar plots generated with the *ggplot2* package [39] in Fig. 2. The analysis of host-symbiont associations (Fig. 3) was conducted using the ComplexHeatmap package (v. 2.14.0) [40]. In addition, the phylogeny of host-symbiont relationships (Fig. 4) was assessed using the Maximum Likelihood (ML) method in MEGA v.11 [41].

**Fig. 5:**
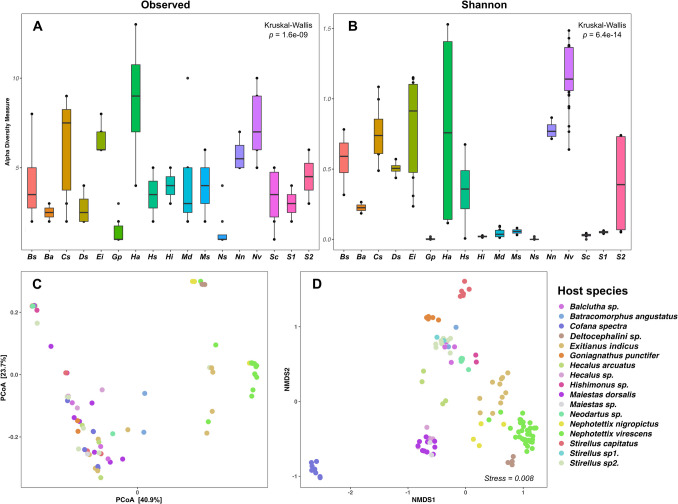
Alpha and Beta diversity of bacterial communities associated with 17 cicadellid species. **A** Boxplots of observed ASV richness and **B** Shannon diversity index show alpha diversity across different host species. **C** Principal Coordinate Analysis (PCoA) based on unweighted *UniFrac* distances and **D** NMDS based on *Bray–Curtis* distances depict beta diversity patterns among individual samples grouped by host species. Abbreviations on the *x-axis* correspond to the following host species: *Balclutha* sp. (Bs)*, Batracomorphus angustatus* (Ba), *Cofana spectra* (Cs)*, Deltocephalini* sp. (Ds), *Exitianus indicus* (Ei)*, Goniagnathus punctifer* (Gp)*, Hecalus arcuatus* (Ha)*, Hecalus* sp. (Hs)*, Hishimonus* sp. (Hi)*, Maiestas dorsalis* (Md)*, Maiestas* sp. (Ms), *Neodartus* sp. (Ns)*, Nephotettix nigropictus* (Nn)*, Nephotettix virescens* (Nv)*, Stirellus capitatus* (Sc)*, Stirellus* sp1. (S1)*,* and *Stirellus* sp2. (S2)

The analysis of bacterial diversity was normalized by rarefying the data to a sequencing depth of 1,184 reads per sample using the ‘*rarefy_even_depth’* function in *phyloseq;* a threshold was chosen to maximize samples’ retention while standardizing coverage. Alpha (α) diversity indices, including observed (*Obs*) ASV and Shannon index, were calculated using the *‘estimate_richness’* function in the *vegan* package (v. 2.6.4) [42]. Statistical comparisons of α-diversity among host species were conducted to estimate microbial richness and evenness. The *Kruskal–Wallis* test was used to compare group distributions, specifically for *Nephotettix virescens*. Beta (β) diversity was evaluated using Principal Coordinate Analysis (PCoA) based on unweighted-*UniFrac* distances and non-metric multidimensional scaling (NMDS) based on *Bray–Curtis* dissimilarity matrices, using the *‘ordinate’* function in *phyloseq* (Fig. 5). Differences in community structure among host species were tested using permutational multivariate analysis of variance (PERMANOVA) with the ‘*adonis2’* function in *vegan*. Pairwise comparisons among host species were performed using the ‘*pairwise.adonis2’* function.

**Fig. 6:**
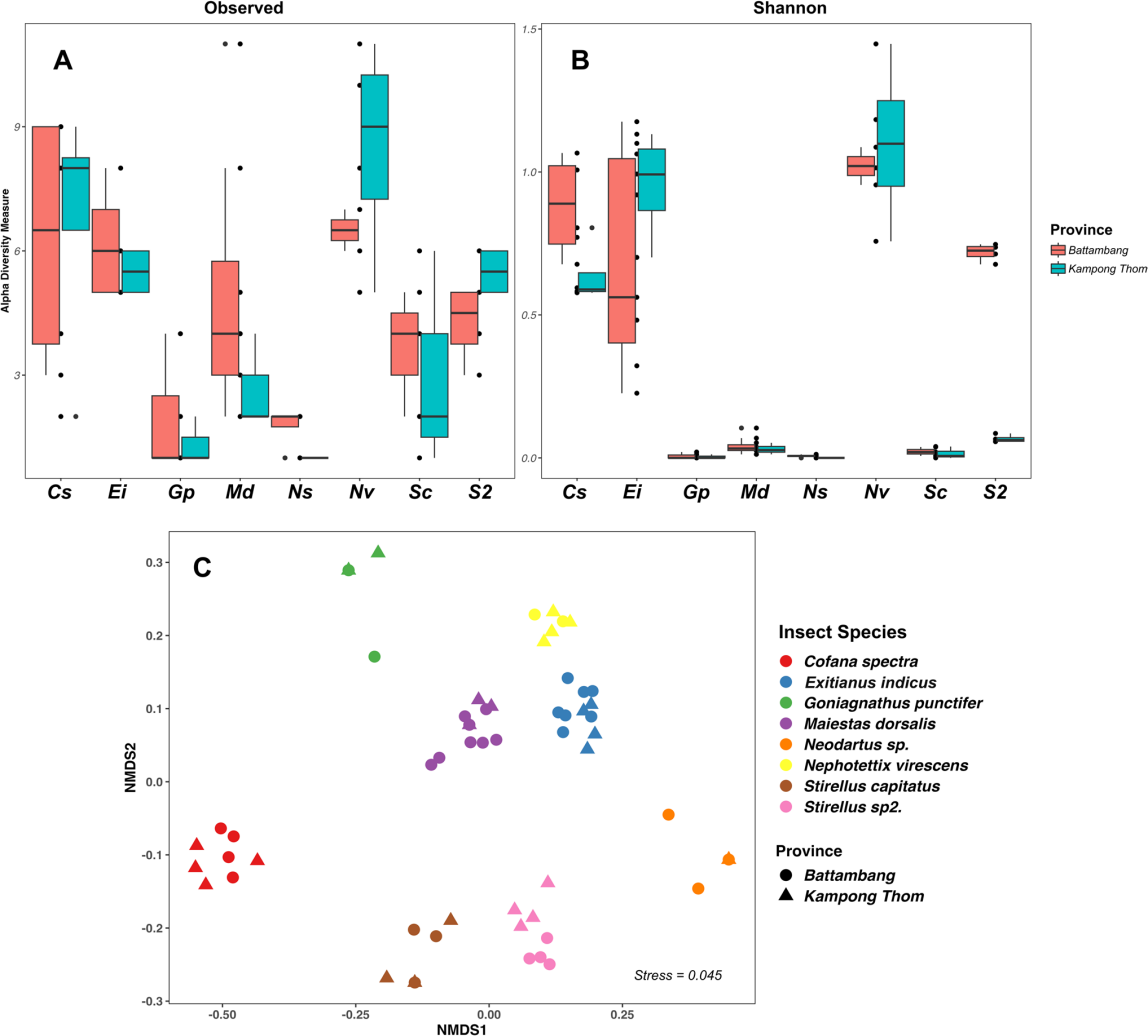
Bacterial communities from different locations (Battambang and Kampong Thom) across eight cicadellid species. Boxplots show alpha diversity comparison between locations: **A** Observed richness and **B** Shannon diversity index. **C** NMDS plot based on *Bray–Curtis* distances illustrates beta diversity patterns between locations across cicadellid species. The abbreviations on the *x-axis* (A and B) represent the following host species: *Cofana spectra* (Cs), *Exitianus indicus* (Ei)*, Goniagnathus punctifer* (Gp)*, Maiestas dorsalis* (Md), *Neodartus* sp. (Ns)*, Nephotettix virescens* (Nv)*, Stirellus capitatus* (Sc)*,* and *Stirellus* sp2. (S2)

To assess the geographic variation in symbiotic community composition between Battambang and Kampong Thom provinces, eight species of rice-associated leafhoppers (*Cofana spectra, Goniagnathus punctifer, Maiestas dorsalis, Nephotettix nigropictus, N. virescens, Neodartus* sp., *Stirellus capitatus*, and *Stirellus* sp2.) were stratified by collection sites in the TSL floodplain. These two provinces were selected due to their geographic separation across the TSL, their significance as major rice production areas in Cambodia, and their provision of a sufficient and balanced sampling size for robust comparative analysis. α-diversity indices were compared between the two provinces using the *Kruskal–Wallis* test, while β-diversity was analyzed via NMDS based on the *Bray–Curtis* dissimilarity matrix, with PERMANOVA applied to assess differences in community structure clustering between provinces (Fig. 6).

**Fig. 7:**
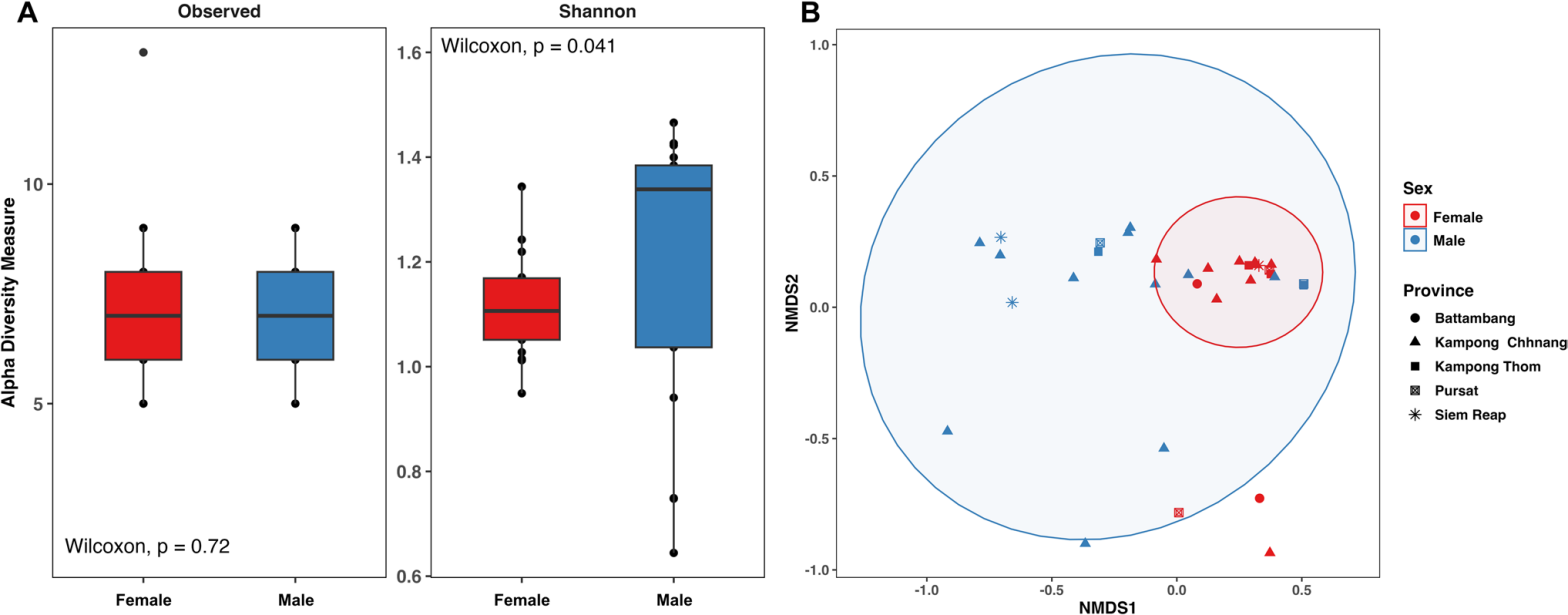
Bacterial communities associated with *Nephotettix virescens*. **A** Alpha diversity comparison between males and females, indicating a higher diversity for males based on the Shannon diversity index, but not for the Observed richness. **B** NMDS plot based on *Bray–Curtis* distances depicts beta diversity patterns between males and females across different locations (provinces), showing differences between sexes, but not between provinces. Additional patterns are provided in the supplementary materials (Figure S4)

**Fig. 8:**
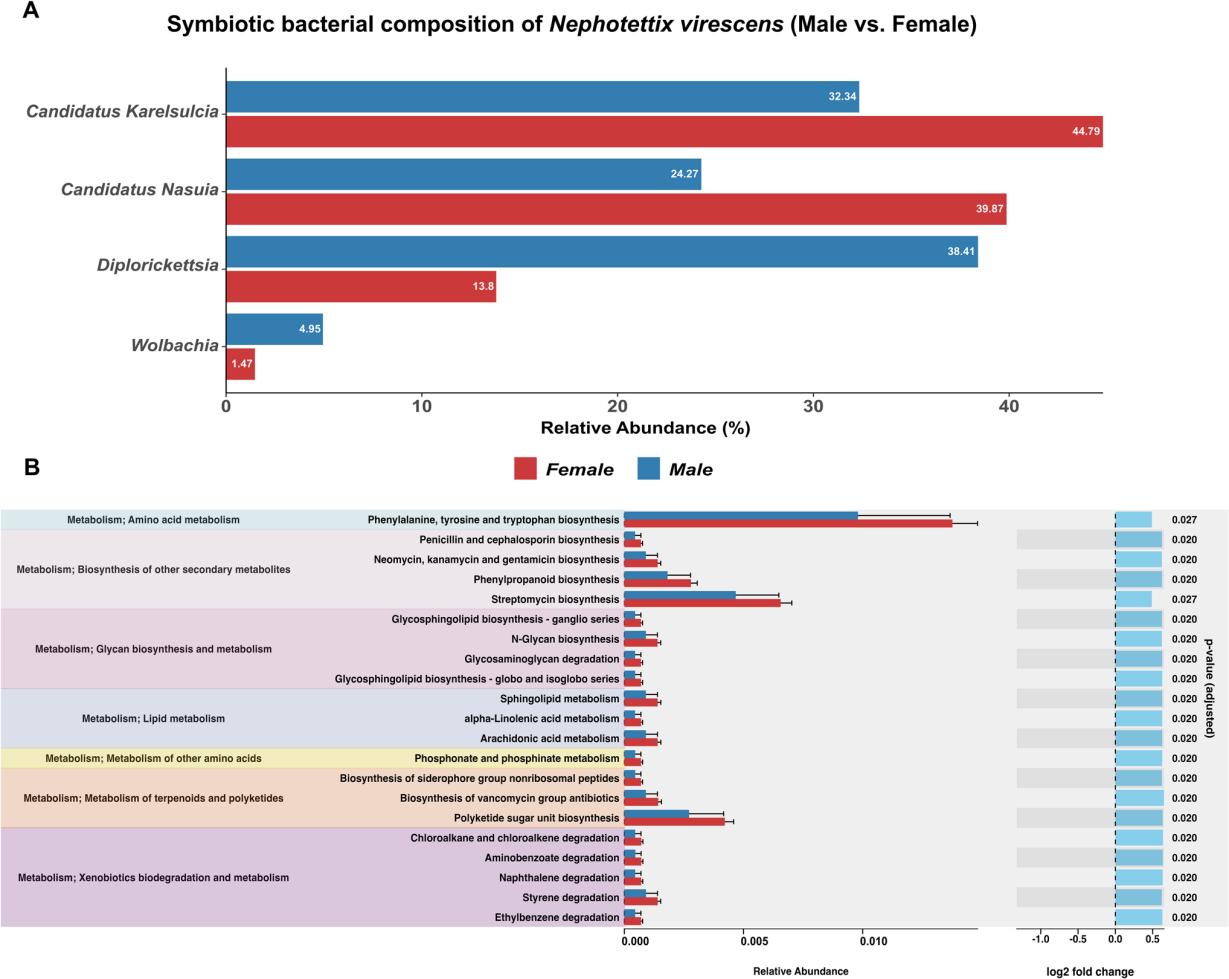
Key endosymbionts and functional pathways differentiating male and female green leafhoppers, *Nephotettix virescens*. **A** Relative abundance (%) of symbiotic bacterial taxa, highlighting the most influential symbionts contributing to differences in bacterial community profiles between male and female of *N. virescens*. The plot displays the top three taxa with the highest score (mean decrease in accuracy/Gini; Figure S4, Table S4). **B** Functional prediction using *PICRUSt2* reveals the relative abundance (with standard error) of significantly different (*p* < 0.05) KEGG Orthology (KO) pathways between male and female, based on the KEGG enzyme and pathway database

To investigate the distribution patterns, focus on Locations (provinces), Season (rainy vs. dry), and Sex-associated differences, a subset of samples from *Nephotettix virescens* was selected for this study. The species was chosen due to its dominant representation in the samples (male vs. female) and its significance as a major pest insect in the region. Community structure differences based on season (Figure S4) and sex-associated (Fig. 7) of *N. virescens* were calculated and visualized as described above. To identify microbial taxa associated with males and females of *N. virescens*, a Random Forest (RF) classification approach was constructed using the *randomForest* package (v. 4.7–1.1) in R [43]. Taxa importance was determined based on the Mean Decrease in Gini Index, with the top-rank ASVs visualized and summarized (Figure S4). Additionally, functional prediction of microbial communities was performed using *PICRUSt2* [44], and KEGG Orthology (KO) pathway annotations were visualized using the *ggpicrust2* R package [45]. Pathway profiles were compared to explore functional differences between males and females of host species (Fig. 8).

## Results

### Bacterial Composition Associated with Cicadellidae Insects

After quality control, we obtained a total of 1,513,348 raw sequence reads from the cicadellid hosts. Following sequence filtering, 1,127,698 high-quality reads were obtained, with an average of 8950 reads per sample (Table S2). The average length of assembly paired-end sequences of the 16S rRNA gene ranged from 402 to 429 bp. All high-quality reads were assigned into 98 ASVs (Table S3). The raw sequence dataset was deposited in the European Nucleotide Archive (ENA) under accession number PRJEB87203.

A total of 98 bacterial ASVs were annotated to four phyla, six classes, 18 orders, 29 families, and 33 genera. Among these, 69.48% ASVs were classified as Bacteroidota, making it the most dominant phylum, followed by Proteobacteria (30.18%). Notably, no ASVs belonging to the phylum *Mycoplasmatota* (including *phytoplasma*) were detected in any of the sampled leafhoppers. At the family level, Blattabacteriaceae (69.47%) and Oxalobacteraceae (14.14%) were the most dominant. At the genus level, the primary endosymbiont *Karelsulcia* was predominant in almost all samples, with an average relative abundance of 69.47%. Three other genera—*Nasuia* (14.14%)*, Diplorickettsia* (7.78%), and *Wolbachia* (5.78%)—also showed relative abundance greater than 1% (Fig. 2; Table 1).

**Table 1:** Symbiotic bacteria associated with Cicadellidae hosts of 17 species in this study and the number of reads in average (per sample) and the relative abundance (%) of eight major symbionts in the host species

Subfamily	Host species	*n*	*Symbionts*								
			***Karelsulcia***	***Nasuia***	***Sodalis***	***Arsenophonus***	***Wolbachia***	***Rickettsia***	***Diplorickettsia***	**Ca.* Lariskellla***	***Others***
**Cicadellinae**	*Cofana spectra*	8	7384.38 (74.57%)		538.38 (5.44%)		820.50 (8.29%)	203.125 (2.05%)		52.50 (0.53%)	904.00 (9.13%)
**Iassinae**	*Batracomorphus angustatus*	2	6093.50 (94.42%)						356.50 (5.52%)		3.50 (0.04%)
**Deltocephalinae**	*Goniagnathus punctifer*	7	7126.57 (99.96%)								3.14 (0.04%)
	*Exitianus indicus*	11	3428.18 (32.67%)	2151.55 (20.50%)		703.64 (6.70%)	2687.82 (25.61%)				1523.64 (14.52%)
	*Balclutha* sp.	4	5024.75 (78.11%)				1139.75 (17.72%)				268.25 (4.17%)
	*Nephotettix nigropictus*	5	3066.00 (48.14%)	2675.20 (42.01%)					624.80 (9.81%)		2.60 (0.04%)
	*Nephotettix virescens*	34	5160.41 (41.95%)	4216.15 (34.27%)		0.09 (0.001%)	113.85 (0.93%)		2792.24 (22.70%)		18.29 (0.15%)
	*Neodartus* sp.	8	8661.75 (99.96%)								3.25 (0.04%)
	*Hishimonus* sp.	2	12,392.00 (99.90%)								12.50 (0.10%)
	*Maiestas dorsalis*	12	6119.58 (99.80%)								12.17 (0.20%)
	*Maiestas* sp.	2	4535.50 (39.08%)								7069.50 (60.92%)
	*Hecalus arcuatus*	4	2972.00 (82.71%)		466.25 (12.98%)					149.25 (4.15%)	5.75 (0.16%)
	*Hecalus* sp.	4	3208.25 (81.02%)				749.50 (18.93%)				2.00 (0.05%)
	*Deltocephalini* sp.*	4	1424.00 (21.13%)	5315 (78.85%)							1.75 (0.03%)
	*Stirellus capitatus*	7	2896.43 (99.82%)								5.14 (0.18%)
	*Stirellus* sp1	4	7922.25 (99.93%)								5.50 (0.07%)
	*Stirellus* sp2	8	7213.50 (61.20%)				4557.38 38.67%)				15.50 (0.13%)

* —* unidentified species*

*n — number of samples per species*

We observed that *Karelsulcia* was the dominant symbiont, with an average relative abundance of 99.89%, in six cicadellid leafhoppers: *Goniagnathus punctifer, Neodartus* sp., *Hishimonus* sp., *Maiestas dorsalis, Stirellus capitatus*, and *Stirellus* sp1., all belonging to the subfamily Deltocephalinae. Interestingly, the co-obligate symbiont *Nasuia* was found only in the following cicadellid hosts: *Exitianus indicus* (20.50%), *Nephotettix nigropictus* (42.01%), *N. virescens* (34.27%), and unidentified *Deltocephalini* sp. (78.85%). *Sodalis*, a recently evolved co-symbiont alongside *Karelsulcia, was* detected only in *Cofana spectra* (5.44%) and *Hacalus arcuatus* (12.98%). In addition, *Wolbachia* was associated with the following hosts: *Cofana spectra* (8.29%), *Exitianus indicus* (25.61%), *Balclutha* sp. (17.72%), *Hecalus* sp. (18.93%), *Stirellus* sp2. (38.67%), and *Nephotettix virescens* (less than 1%). The symbiotic bacterium *Diplorickettsia* was detected in *N. virescens* (22.70%), *N. nigropictus* (9.81%), and *Batracomorphus angustatus* (5.52%).

### Symbionts Associating with Cicadellidae

The analysis of primary and secondary symbionts based on the amplicon sequence variants (ASVs) was conducted on 17 Cicadellidae insects collected from the TSL floodplains. The obligate endosymbiont *Karelsulcia* was found to be the most dominant bacterium associated with the cicadellid hosts and was consistently present across all species. However, the composition of co-obligate and secondary symbionts varied significantly among host species (Fig. 3; Table S5). Seven species—including *Maiestas dorsalis* and *Maiestas* sp. (tribe Deltocephalini), *Goniagnathus punctifer* (Gonignathini), *Hishimonus* sp. (Opisiini), *Neodartus* sp. (Penthimiini), and *Stirellus capitatus* and *Stirellus* sp1. (Stenometopiini)—harbored only the obligate symbiont *Karelsulcia*. The co-obligate symbiont *Nasuia* was detected in species from the tribe Chiasmini—namely *Nephotettix nigropictus*, *N. virescens*, and *Exitianus indicus*—as well as in an unidentified species within the tribe Deltocephalini in the subfamily Deltocephalinae. Secondary symbionts, including *Sodalis* and *Ca.* Lariskella, were identified in some samples of *Cofana spectra* (Cicadellini) and *Hecalus arcuatus* (Hecalini). *Arsenophonus* appeared at low frequency, being found only in *E. indicus*, while *Rickettsia* was detected in *C. spectra*. A close relative of *Rickettsia*, *Diplorickettsia*, was present in most samples of *Batracomorphus angustatus* (Batracomorphini) and *N. virescens*, but only a single individual of *N. nigropictus*. Additionally, *Wolbachia* was detected in samples of *Balclutha* sp. (Hecalini) and *E. indicus*, and to a lesser extent in *C. spectra*, *Hecalus* sp., *N. virescens*, and *Stirellus* sp2.

### Microbiota Diversity Across Cicadellidae Species

Alpha diversity of Cicadellidae species was assessed using observed richness (*Obs*) and the Shannon–Wiener diversity index (*H*). We observed higher bacterial richness in *Hecalus arcuatus* based on *Obs*, while *Nephotettix virescens* showed greater bacterial diversity according to the *H* index (Fig. 5A, B). The *Kruskal–Wallis* rank test revealed significant differences in bacterial diversity among cicadellid species (*p-*value < 0.05). Pairwise comparisons using the *Wilcoxon* rank sum test showed significant differences (*p-*value < 0.05) in both *Obs* richness and *H* index between certain species pairs (Table S6). Interestingly, pairwise comparisons of bacterial composition between *Nephotettix virescens* and other host species showed that *N. virescens* had a statistically significant more diverse microbiota, except in comparison with *Hecalus arcuatus*.

Beta diversity of bacterial communities was analyzed to determine whether the microbial composition and structure differed across the 17 cicadellid species. Diversity metrics were calculated using both phylogenetic-based distance (unweighted *UniFrac*) and compositional dissimilarity (*Bray–Curtis*), and visualized through PCoA and NMDS ordinations (Fig. 5C, D). While the PCoA plot did not show highly distinct differences among species, the NMDS ordination revealed clustering patterns between species. PERMANOVA analysis confirmed significant differences in bacterial communities among species for both *Bray–Curtis* distance (*R*^2^ = 0.87, *F* = 42.53, *p* = 0.001) and unweighted *UniFrac* (*R*^2^ = 0.62, *F* = 10.50, *p* = 1e-04). Additionally, pairwise PERMANOVA comparisons (Table S7) revealed significant differences (*p-*value < 0.05) in several species’ pairs. Based on *Bray–Curtis* dissimilarity, significant differences were observed in comparisons involving *Cofana spectra, Exitianus indicus, Neodardus* sp., *Nephotettix virescens*, and *Stirellus capitatus*. In contrast, comparisons using unweighted *UniFrac* distances showed that *Nephotettix virescens* differed significantly from nearly all other species, with the exception of its comparison with *N. nigropictus,* which was not statistically significant.

### Geographic variation in symbiotic communities across two provinces

Eight Cicadellidae species: *Cofana spectra, Goniagnathus punctifer, Maiestas dorsalis, Nephotettix nigropictus, N. virescens, Neodartus* sp., *Stirellus capitatus*, and *Stirellus* sp2., were collected from two locations: Battambang and Kampong Thom. *Wolbachia* was exclusively detected in *Stirellus* sp2. from Battambang, while *Lactococcus* was only found in *Cofana spectra* from the same province. Four species (*Goniagnathus punctifer, Maiestas dorsalis, Neodartus* sp., and *Stirellus capitatus*) were consistently dominated by *Karelsulcia* (>99%) across both locations. Differences in bacterial composition between the two provinces are illustrated in Figure S3.

Alpha diversity (*Obs* richness and Shannon index) showed no significant differences in bacterial communities between Battambang and Kampong Thom for most species (Fig. 6). Analysis of the Wilcoxon test comparison grouped by species confirmed that there were no significant inter-provincial differences, except for *Stirellus* sp2. (*p* = 0.028). The ordination (Fig. 6C) showed clear clustering and separation of species. PERMANOVA results (Table 2B) indicated that bacterial community composition was primarily structured by host species identity (*R*^2^ = 0.90, *F* = 77.23, *p* = 0.001). Although provinces had a statistically significant effect (*R*^2^ = 0.007, *F* = 4.58, *p* = 0.001), its contribution to overall variation was minor, suggesting that the influence of geographic location on microbial communities differs across cicadellid species.

**Table 2:** Summary of PERMANOVA models of *Bray–Curtis* distances: **A** The effects of two locations on microbial communities across cicadellid host species and **B** variation in microbial community structure within *Nephotettix virescens*

Variables	Sums of squares	*R*^*2*^	F model	*p value*
***(A) Two locations effect on cicadellid host species***				
HostSpecies	25.342	0.900	77.233	***0.001***
Province	25.134	0.007	4.584	***0.001***
Residual	2.578	0.091		
Total	28.135	1		
***(B) Nephotettix virescens***				
Sex	0.483	0.142	5.111	***0.001***
Season:Province	0.402	0.118	0.708	0.772
Residual	2.458	0.723		
Total	3.397	1		

Significant *p*-values (< 0.05) are shown in bold and italic

### Distribution Patterns of Bacterial Communities in Nephotettix virescens

The bacterial composition of *Nephotettix virescens* revealed that *Karelsulcia* (38.56%), *Nasuia* (32.07%), and *Diplorickettsia* (26.10%) were the dominant taxa across samples (Figure S3). When comparing bacterial compositions between male and female, clear differences in the relative abundances for the symbiotic bacteria, *Karelsulcia, Nasuia, Diplorickettsia*, and *Wolbachia* were observed (Figure S4). The analysis of alpha diversity based on *Obs* richness and *Shannon H* index across sex, locations, and season is presented in Figs. 7A, S4. A significant difference was found only in the *Shannon* (*H*) index between male and female of *N. virescens* (*p* < 0.05). No significant differences were detected in bacterial communities across different locations or season. For beta diversity, PERMANOVA analysis based on *Bray–Curtis* distance showed that sex had a significant effect on the bacterial community structure of *N. virescens* (*R*^2^ = 0.14, *F* = 5.11, *p* = 0.001). This suggests that males and females harbor distinct microbiota. However, neither province, season, nor any interactions among the variables significantly influenced microbial composition (Table 2, Fig. 7B).

### Core Microbiota and Functional Analysis of Nephotettix virescens

In *Nephotettix virescens*, four abundant symbiotic bacteria—*Karelsulcia, Nasuia, Diplorickettsia*, and *Wolbachia*—showed a difference in bacterial composition between males and females (Fig. 8A). In addition, Random Forest (RF) classification, based on the Mean Decrease in Gini Index, identified the top three core bacterial taxa contributing to these sex-associated differences*.* These included *Karelsulcia* (ASV_13) and two *Diplorickettsia* variants (ASV_8 and ASV_12), which exhibited distinct patterns between male and female insects (Figure S4). The ranking of important microbial taxa based on their Mean Decrease Gini scores is presented in Table S4. To explore the potential functional roles of the bacterial community of *N. virescens*, functional predictions were conducted using *PICRUSt2*. Putative functional profiles were inferred from 16S rRNA gene data by mapping identified ASVs to the KEGG Orthology (KO), KEGG Enzyme, and KEGG pathway database (Fig. 8B). The most abundant KO categories were associated with key metabolic processes: amino acids metabolism (phenylalanine, tyrosine, and tryptophan), secondary metabolite biosynthesis (streptomycin), and polyketide sugar unit biosynthesis. Among the top 21 significantly different (*p* < 0.05) KEGG KO categories, notable sex-associated variations were observed. Overall, male *N. virescens* exhibited a lower abundance of predicted functional pathways compared to females.

## Discussion

### Symbiotic Diversity of Cicadellidae Insects

The study provides a comprehensive characterization of the symbiotic bacterial communities of rice-associated leafhoppers (Cicadellidae) from the floodplains of Tonle Sap Lake in Cambodia. The symbiotic diversity of the 17 cicadellid species was dominated by the primary endosymbiont *Candidatus* Karelsulcia muelleri (Fig. 2, Table 1). Our findings underscore the importance of Cicadellidae species in shaping microbial diversity, with obligate symbiont *Karelsulcia* emerging as the most dominant symbiont across most cicadellid species [29, 46, 47] and its co-obligate symbiont *Nasuia,* present particularly in the subfamily Deltocephalinae. In addition to obligate symbionts, we detected several secondary symbionts (*Sodalis*, *Wolbachia*, *Arsenophonus, Rickettsia*, and *Diplorickettsia*), which are known to affect host fitness, pathogen transmission, and insecticide resistance in other insect systems [9, 21]. However, we did not detect any ASVs belonging to the phylum Mycoplasmatota (*phytoplasma*), despite their known association with rice agroecosystems and transmission by rice-associated leafhopper species [1, 7, 47, 48]. This absence may reflect the low prevalence of *phytoplasma* infection in the sampled populations. Among these cicadellid hosts, *Hecalus arcuatus* exhibited the highest bacterial richness, while *Nephotettix virescens* showed the greatest diversity based on the *Shannon* index (Fig. 5A, B). This elevated diversity in *N. virescens* may be attributed to its status as a rice pest that feeds on a broader range of host plants or adapts to various environments, potentially increasing its exposure to a more diverse microbial community [49]. Bacterial communities differed significantly among cicadellid species, explaining 62% and 87% of the variation in community structure based on PERMANOVA using unweighted *UniFrac* and *Bray–Curtis* distances, respectively (Table S7). While there are several possible drivers of these interspecific differences—such as host traits, diet, phylogeny, or environmental factors [10, 13, 21, 50]—we acknowledge that these factors were not formally tested in this study and warrant further investigation in future research.

### Primary and Secondary Symbionts of Cicadellidae

*Ca.* Karelsulcia muelleri (*Karelsulcia*) is an obligate primary symbiont widely known for its long-standing association with sap-feeding insects in the suborder Auchenorrhyncha, where it plays an essential role in nutrient provisioning [13–17, 48, 51, 52]. A recent phylogenetic study of *Karelsulcia* from 52 insect species across three Auchenorrhyncha superfamilies—Cicadoidea, Cercopoidea, and Membracoidea—revealed consistent evolutionary patterns, highlighting the stable co-evolution of this symbiont with its hosts [52]. From our 17 rice-associated leafhopper species, the co-obligate symbiont *Ca.* Nasuia deltocephalinicola (*Nasuia*) was detected in four species within the subfamily Deltocephalinae: *Exitianus indicus, Nephotettix nigropictus, N. virescens*, and *Deltocephalini* sp. (Fig. 4). *Nasuia* has previously been reported in several other Deltocephalinae species, including *Nephotettix cincticeps* [19], *Matsumuratettix hiroglyphicus* [47], and *Macrosteles striifons* and *M. sexnotatus* [48]. The majority of leafhoppers in the subfamily Deltocephalinae harbor at least two obligate endosymbionts: *Karelsulcia*, which provides eight of the ten essential amino acids missing from their plant-sap diet, and co-obligate partners such as *Nasuia*, which contribute the remaining two [17, 19, 53]. Interestingly, *Nasuia* was detected in only 5 out of 11 individual samples of *E. indicus*, suggesting the possibility of symbiont replacement, loss, or functional complementation by other bacteria, such as *Arsenophonus* and *Wolbachia*, as shown in Fig. 3 and Table S5. The co-occurrence of such symbionts has previously been linked to insecticide resistance in *Nilaparvata lugens* [21].

The common ancestor of the Auchenorrhyncha acquired *Karelsulcia* and β-proteobacterial symbionts over 260 million years ago [13]. However, over evolutionary time, the co-obligate β-proteobacterial symbionts were occasionally lost or replaced in certain lineages, for example, by γ-Proteobacteria such as *Baumannia* in Sharpshooter leafhoppers [14, 53, 54]. In this study, six Deltocephalinae species—*Goniagnathus punctifer*, *Hishimonus* sp., *Maiestas dorsalis, Maiestas* sp.*, Neodartus* sp., *Stirellus capitatus*, and *Stirellus* sp1.—were found to harbor only endosymbiont *Karelsulcia*, with no detectable co-obligate β-proteobacterial symbionts. Previous studies suggest that such species may instead host mycetocyte-associated yeast-like symbionts [13, 52]. It is possible that *Nasuia* has been replaced by yeast-like symbionts in these lineages; however, this hypothesis remains speculative and requires further validation, such as fungal community profiling using the 18S rRNA gene.

We observed several secondary symbionts—*Arsenophonus, Ca *Lariskella*, Diplorickettsia, Rickettsia, Sodalis*, and *Wolbachia*—as shown in Figs. 3 and 4*.* Notably, *Sodalis* and *Ca.* Lariskella showed strong associations with specific cicadellid hoppers (*Cofana spectra* and *Hecalus arcuatus*, respectively), suggesting potential host-symbiont coevolution and adaptation. *Sodalis* has been reported to function as a co-obligate symbiont in the long-tailed mealybugs *Pseudococcus longispinus* [55] and spittlebug *Philaenus spumarius* [51]. Meanwhile, *Ca.* Lariskella has been described as a facultative symbiont found in stinkbugs, fleas, and ticks [56]. Interestingly, the γ-Proteobacterium *Arsenophonus* was only detected in *Exitianus indicus*, although it has been reported to be widespread across arthropod species [57]. It has been implicated in promoting whitefly adaptation to specific host plants [58] and in protecting arthropods from parasitoid attacks [59].

Other co-occurring endosymbionts—*Diplorickettsia, Rickettsia*, and *Wolbachia*—have been identified in various Deltocephalinae species [13, 48, 60]. *Diplorickettsia* was dominant in *Batracomorphus angustatus* and *N. virescens*, with a single detection in *N. nigropictus* in this study (Fig. 3). It may complement *Nasuia* in the *N. virescens* host or replace the co-obligate symbiont in *B. angustatus,* potentially fulfilling the role of producing all essential amino acids. Additionally, *Rickettsia* was detected only in *C. spectra* and may act in synergy with *Sodalis* to supplement amino acid synthesis. In contrast, *Wolbachia,* an intracellular symbiont found in between 20 and 76% of arthropod species [61], was detected in six cicadellid species in our study (Fig. 4). *Wolbachia* may play a compensatory role, potentially replacing *Nasuia* in *Balclutha* sp. and *Hecalus* sp., or complementing *Nasuia* in *E. indicus.* Overall, these findings suggest a dynamic interplay between primary (obligate) and secondary symbionts in cicadellid hosts.

### Limited Geographic Influence on Microbial Composition in Cicadellidae

The influence of geographic origin on microbial diversity of the cicadellid hosts was generally minimal across most species, with the exception of *Stirellus* sp2., which showed significant differences observed between populations from the Battambang and Kampong Thom provinces (Fig. 6). A similar pattern of limited geographic variation in microbial communities was observed in populations of *Macrosteles* leafhoppers [9]. In contrast, research on the glassy-winged sharpshooter, *Homalodisca vitripennis,* indicated that bacterial communities were strongly influenced by geographic location [62]. Notably, *Wolbachia* was predominantly detected only in the *Stirellus* sp2. population from Battambang (Figure S3), suggesting that the dominance of specific symbionts may influence the overall microbiome composition within leafhopper populations. *Wolbachia* is well known as a manipulator of host reproduction [21] and may contribute to its spread within host populations through maternal inheritance. These patterns of variability may be driven by local ecological factors, such as plant availability and microclimatic conditions, which vary across the TSL floodplain regions and seasons [22]. Although the overall community structure showed a statistically significant effect of location (Table 2A), it accounted for only a small portion of the variation (0.7%) based on PERMANOVA analysis, indicating a relatively weak geographic influence. These findings suggest that, while location can shape microbial communities, its impact is strongly dependent on host species.

### Difference Patterns of Symbionts in Nephotettix virescens

Although we did not observe significant variation in between provinces and seasons, it is plausible that male and female *Nephotettix virescens* exhibit differences in their microbial composition, accounting for approximately 14% of the variation (Fig. 7, Table 2B). These differences may influence various aspects of their biology, including nutrition, immunity, and reproduction [4]. Distinct sex-associated differences in microbial composition were observed, with male and female *N. virescens exhibiting* distinct relative abundances of *Karelsulcia*, *Nasuia*, *Diplorickettsia*, and *Wolbachia* (Fig. 8A). This is a novel finding and warrants further investigation, as microbial symbionts have been shown to affect reproductive success, fitness, and disease transmission in other insect species [16]. Sex-associated differences in microbial communities of *N. virescens* were primarily driven by the important endosymbionts *Karelsulcia* and two *Diplorickettsia* variants, as revealed by Random Forest analysis (Figure S4). *Karelsulcia* and *Nasuia* showed high density in females, suggesting that they proliferate and migrate to the ovaries of female insects, as shown in a similar study in the cicada *Meimuna mongolica* [63]. However, *Diplorickettsia* and *Wolbachia* showed lower abundance in females, which could be due to competition with other microorganisms, such as the symbionts *Karelsulcia* and *Nasuia,* potentially reducing their bacterial densities.

In addition, the functional prediction of microbial communities using *PICRUSt2* further revealed sex-associated variations in metabolic pathways, particularly in amino acid metabolism and secondary metabolite biosynthesis (Fig. 8B). The dominance of the amino acid biosynthesis pathway was observed in females of *N. virescens*, a pattern commonly reported in microbiome studies where such enrichment is linked to reproductive investment in female insects. For example, Tyrosine and Phenylalanine produced by endosymbionts are shown to be indispensable for insect growth and survival in several species [64]. Moreover, Tyrosine pathway regulation was reported as host-mediated in pea aphid symbiosis during late embryonic and early larval development [65]. However, it is worth noting that the results of the functional analysis presented in this study are limited to the information contained in the KEGG database. Despite this apparent limitation, our findings demonstrate a clear pattern in both the composition and potential functions of the microbial communities, which may be linked to the activity of endosymbionts. Further research focusing on *N. virescens* is needed to explore these potential sex-based microbial variations.

## Conclusions

The study provides preliminary results on the symbiotic diversity of 17 rice-associated (Cicadellidae) from the floodplains of the Tonle Sap Lake, Cambodia. The findings emphasize the dominance of *Karelsulcia* as the primary endosymbiont across all species, highlighting its essential role in supporting the nutritional needs of their hosts. In addition to *Karelsulcia*, a co-obligate *Nasuia* and secondary symbionts such as *Sodalis, Wolbachia, and Diplorickettsia* were identified, with notable differences in their distribution and abundance among species. Geographic location had minimal influence on microbial diversity, although some species exhibited geographic-specific shifts in symbiotic profiles. No significant variation was observed in the microbial composition of *Nephotettix virescens* across seasons and locations*.* However, sex-associated differences were evident in *N. virescens*, with females exhibiting a higher abundance of *Karelsulcia*, while *Diplorickettsia* was less abundant, likely due to competition with other dominant symbionts. Overall, the microbial communities of these leafhoppers reflect a dynamic interplay between obligate and secondary symbionts, potentially influencing host fitness, immunity, and environmental adaptation. Further studies, incorporating functional genomics and host-symbiont interactions analysis, are crucial to understanding the ecological roles and evolutionary dynamics of these microbial communities.

## Supplementary Information

Below is the link to the electronic supplementary material.

ESM 1 (DOCX 86.1 KB)

ESM 2 (XLSX 27.9 KB)

ESM 3 (DOCX 0.98 MB)

## Data Availability

The raw bacterial 16S rRNA (V3–V4) gene sequence reads were deposited in the European Nucleotide Archive (ENA accession *PRJEB87203*).
