# Supplementary material for: Symbiont Diversity of Rice-Associated Leafhoppers (Cicadellidae) in the Tropical Floodplains of the Tonle Sap Lake, Cambodia
Source: Microb Ecol. 2025 Oct 17;88:109. doi: 10.1007/s00248-025-02619-9 (PMC12534257; doi:10.1007/s00248-025-02619-9)
Supplement: Supplementary file 1 [file 248_2025_2619_MOESM1_ESM.docx]

***Symbiont diversity of rice-associated leafhoppers (Cicadellidae) in the tropical floodplains of Tonle Sap Lake, Cambodia***

**Sophany Phauk^1,2^*, Lorenzo Assentato^2^, Sopha Sin^3^, Onnorong Uk^1^, Sophorn Hap^1^ and Olle Terenius^2^**

^1^	Department of Biology, Faculty of Science, Royal University of Phnom Penh, Cambodia

^2^	Department of Cell and Molecular Biology, Microbiology, Uppsala University, Uppsala, Sweden

^3^	Centre for Biodiversity Conservation, Faculty of Science, Royal University of Phnom Penh, Cambodia

*	Corresponding author

Email address: [sophany.phauk@icm.uu.se](mailto:sophany.phauk@icm.uu.se)

**Table S1:** Metadata and information of the dataset

| **SampleID** | **Insect Species** | **Sex** | **Location** | **Season** | **Coordinates** | **Col. site** | **Collection date** |
| --- | --- | --- | --- | --- | --- | --- | --- |
| TS01 | *Nephotettix virescens* | M | Kampong Chhnang | Rainy | 12°11'11.50"N; 104°39'47.30"E | NSP01 | September-2020 |
| TS02 | *Nephotettix virescens* | M | Kampong Chhnang | Rainy | 12°11'11.50"N; 104°39'47.30"E | NSP01 | September-2020 |
| TS03 | *Nephotettix virescens* | F | Kampong Chhnang | Rainy | 12°11'11.50"N; 104°39'47.30"E | NSP01 | September-2020 |
| TS04 | *Nephotettix virescens* | F | Kampong Chhnang | Rainy | 12°11'11.50"N; 104°39'47.30"E | NSP01 | September-2020 |
| TS05 | *Nephotettix virescens* | M | Pursat | Rainy | 12°31'45.20"N; 104°10'58.49"E | NSP03 | September-2020 |
| TS06 | *Nephotettix virescens* | M | Pursat | Rainy | 12°31'45.20"N; 104°10'58.49"E | NSP03 | September-2020 |
| TS07 | *Nephotettix virescens* | F | Pursat | Rainy | 12°31'45.20"N; 104°10'58.49"E | NSP03 | September-2020 |
| TS08 | *Nephotettix virescens* | F | Pursat | Rainy | 12°31'45.20"N; 104°10'58.49"E | NSP03 | September-2020 |
| TS09 | *Nephotettix nigropictus* | M | Battambang | Rainy | 13°13'9.87"N; 103° 6'5.43"E | NSP05 | September-2020 |
| TS10 | *Nephotettix nigropictus* | F | Battambang | Rainy | 13°13'9.87"N; 103° 6'5.43"E | NSP05 | September-2020 |
| TS11 | *Nephotettix virescens* | F | Battambang | Rainy | 13°13'9.87"N; 103° 6'5.43"E | NSP05 | September-2020 |
| TS12 | *Nephotettix virescens* | M | Siem Reap | Rainy | 13°19'59.4"N; 104°00'16.5"E | NSP08 | September-2020 |
| TS13 | *Nephotettix virescens* | M | Siem Reap | Rainy | 13°19'59.4"N; 104°00'16.5"E | NSP08 | September-2020 |
| TS14 | *Nephotettix virescens* | F | Siem Reap | Rainy | 13°19'59.4"N; 104°00'16.5"E | NSP08 | September-2020 |
| TS15 | *Nephotettix virescens* | F | Siem Reap | Rainy | 13°19'59.4"N; 104°00'16.5"E | NSP08 | September-2020 |
| TS16 | *Nephotettix virescens* | M | Kampong Thom | Rainy | 12°53'15.2"N; 104°36'18.8"E | NSP09 | September-2020 |
| TS17 | *Nephotettix virescens* | M | Kampong Thom | Rainy | 12°53'15.2"N; 104°36'18.8"E | NSP09 | September-2020 |
| TS18 | *Nephotettix virescens* | F | Kampong Thom | Rainy | 12°53'15.2"N; 104°36'18.8"E | NSP09 | September-2020 |
| TS19 | *Nephotettix virescens* | F | Kampong Thom | Rainy | 12°53'15.2"N; 104°36'18.8"E | NSP09 | September-2020 |
| TS20 | *Nephotettix virescens* | M | Kampong Chhnang | Rainy | 12°17'03.1"N; 104°35'14.9"E | NSP02 | November-2019 |
| TS21 | *Nephotettix virescens* | M | Kampong Chhnang | Rainy | 12°17'03.1"N; 104°35'14.9"E | NSP02 | November-2019 |
| TS22 | *Nephotettix virescens* | M | Kampong Chhnang | Rainy | 12°17'03.1"N; 104°35'14.9"E | NSP02 | November-2019 |
| TS23 | *Nephotettix virescens* | F | Kampong Chhnang | Rainy | 12°17'03.1"N; 104°35'14.9"E | NSP02 | November-2019 |
| TS24 | *Nephotettix virescens* | M | Kampong Chhnang | Dry | 12°17'03.1"N; 104°35'14.9"E | NSP02 | January-2020 |
| TS25 | *Nephotettix virescens* | M | Kampong Chhnang | Dry | 12°17'03.1"N; 104°35'14.9"E | NSP02 | January-2020 |
| TS26 | *Nephotettix virescens* | F | Kampong Chhnang | Dry | 12°17'03.1"N; 104°35'14.9"E | NSP02 | January-2020 |
| TS27 | *Nephotettix virescens* | F | Kampong Chhnang | Dry | 12°17'03.1"N; 104°35'14.9"E | NSP02 | January-2020 |
| TS28 | *Nephotettix virescens* | M | Kampong Chhnang | Rainy | 12°17'03.1"N; 104°35'14.9"E | NSP02 | July-2020 |
| TS29 | *Nephotettix virescens* | M | Kampong Chhnang | Rainy | 12°17'03.1"N; 104°35'14.9"E | NSP02 | July-2020 |
| TS30 | *Nephotettix virescens* | F | Kampong Chhnang | Rainy | 12°17'03.1"N; 104°35'14.9"E | NSP02 | July-2020 |
| TS31 | *Nephotettix virescens* | F | Kampong Chhnang | Rainy | 12°17'03.1"N; 104°35'14.9"E | NSP02 | July-2020 |
| TS32 | *Nephotettix virescens* | M | Kampong Chhnang | Rainy | 12°17'03.1"N; 104°35'14.9"E | NSP02 | September-2020 |
| TS33 | *Nephotettix virescens* | M | Kampong Chhnang | Rainy | 12°17'03.1"N; 104°35'14.9"E | NSP02 | September-2020 |
| TS34 | *Nephotettix virescens* | F | Kampong Chhnang | Rainy | 12°17'03.1"N; 104°35'14.9"E | NSP02 | September-2020 |
| TS35 | *Nephotettix virescens* | F | Kampong Chhnang | Rainy | 12°17'03.1"N; 104°35'14.9"E | NSP02 | September-2020 |
| TS36 | *Nephotettix nigropictus* | M | Battambang | Dry | 13°13'9.87"N; 103° 6'5.43"E | NSP05 | January-2020 |
| TS37 | *Nephotettix nigropictus* | M | Battambang | Dry | 13°13'9.87"N; 103° 6'5.43"E | NSP05 | January-2020 |
| TS38 | *Nephotettix nigropictus* | F | Battambang | Dry | 13°13'9.87"N; 103° 6'5.43"E | NSP05 | January-2020 |
| TS39 | *Nephotettix virescens* | F | Battambang | Dry | 13°13'9.87"N; 103° 6'5.43"E | NSP05 | January-2020 |
| TS40 | *Exitiainus indicus* | M | Battambang | Dry | 13°13'9.87"N; 103° 6'5.43"E | NSP05 | January-2020 |
| TS41 | *Exitiainus indicus* | M | Battambang | Dry | 13°13'9.87"N; 103° 6'5.43"E | NSP05 | January-2020 |
| TS42 | *Exitiainus indicus* | M | Battambang | Dry | 13°13'9.87"N; 103° 6'5.43"E | NSP05 | January-2020 |
| TS43 | *Maiestas dorsalis* | M | Battambang | Dry | 13°13'9.87"N; 103° 6'5.43"E | NSP05 | January-2020 |
| TS44 | *Maiestas dorsalis* | F | Battambang | Dry | 13°13'9.87"N; 103° 6'5.43"E | NSP05 | January-2020 |
| TS45 | *Maiestas dorsalis* | M | Battambang | Dry | 13°13'9.87"N; 103° 6'5.43"E | NSP05 | January-2020 |
| TS46 | *Maiestas dorsalis* | F | Battambang | Dry | 13°13'9.87"N; 103° 6'5.43"E | NSP05 | January-2020 |
| TS47 | *Exitiainus indicus* | M | Battambang | Rainy | 13°13'9.87"N; 103° 6'5.43"E | NSP05 | September-2020 |
| TS48 | *Exitiainus indicus* | M | Battambang | Rainy | 13°13'9.87"N; 103° 6'5.43"E | NSP05 | September-2020 |
| TS49 | *Exitiainus indicus* | F | Battambang | Rainy | 13°13'9.87"N; 103° 6'5.43"E | NSP05 | September-2020 |
| TS50 | *Exitiainus indicus* | F | Battambang | Rainy | 13°13'9.87"N; 103° 6'5.43"E | NSP05 | September-2020 |
| TS51 | *Maiestas dorsalis* | M | Battambang | Rainy | 13°13'9.87"N; 103° 6'5.43"E | NSP05 | September-2020 |
| TS52 | *Maiestas dorsalis* | M | Battambang | Rainy | 13°13'9.87"N; 103° 6'5.43"E | NSP05 | September-2020 |
| TS53 | *Maiestas dorsalis* | F | Battambang | Rainy | 13°13'9.87"N; 103° 6'5.43"E | NSP05 | September-2020 |
| TS54 | *Maiestas dorsalis* | F | Battambang | Rainy | 13°13'9.87"N; 103° 6'5.43"E | NSP05 | September-2020 |
| TS55 | *Exitiainus indicus* | M | Kampong Thom | Rainy | 12°53'15.2"N; 104°36'18.8"E | NSP09 | September-2020 |
| TS56 | *Exitiainus indicus* | M | Kampong Thom | Rainy | 12°53'15.2"N; 104°36'18.8"E | NSP09 | September-2020 |
| TS57 | *Exitiainus indicus* | F | Kampong Thom | Rainy | 12°53'15.2"N; 104°36'18.8"E | NSP09 | September-2020 |
| TS58 | *Exitiainus indicus* | F | Kampong Thom | Rainy | 12°53'15.2"N; 104°36'18.8"E | NSP09 | September-2020 |
| TS59 | *Maiestas dorsalis* | M | Kampong Thom | Rainy | 12°53'15.2"N; 104°36'18.8"E | NSP09 | September-2020 |
| TS60 | *Maiestas dorsalis* | M | Kampong Thom | Rainy | 12°53'15.2"N; 104°36'18.8"E | NSP09 | September-2020 |
| TS61 | *Maiestas dorsalis* | F | Kampong Thom | Rainy | 12°53'15.2"N; 104°36'18.8"E | NSP09 | September-2020 |
| TS62 | *Maiestas dorsalis* | F | Kampong Thom | Rainy | 12°53'15.2"N; 104°36'18.8"E | NSP09 | September-2020 |
| TS63 | *Hecalus* sp*.* | M | Kampong Thom | Rainy | 12°53'15.2"N; 104°36'18.8"E | NSP09 | September-2020 |
| TS64 | *Hecalus* sp*.* | M | Kampong Thom | Rainy | 12°53'15.2"N; 104°36'18.8"E | NSP09 | September-2020 |
| TS65 | *Hecalus* sp*.* | F | Kampong Thom | Rainy | 12°53'15.2"N; 104°36'18.8"E | NSP09 | September-2020 |
| TS66 | *Hecalus* sp*.* | F | Kampong Thom | Rainy | 12°53'15.2"N; 104°36'18.8"E | NSP09 | September-2020 |
| TS67 | *Goniagnathus punctifer* | M | Kampong Thom | Rainy | 12°53'15.2"N; 104°36'18.8"E | NSP09 | September-2020 |
| TS68 | *Goniagnathus punctifer* | M | Kampong Thom | Rainy | 12°53'15.2"N; 104°36'18.8"E | NSP09 | September-2020 |
| TS69 | *Goniagnathus punctifer* | F | Kampong Thom | Rainy | 12°53'15.2"N; 104°36'18.8"E | NSP09 | September-2020 |
| TS70 | *Goniagnathus punctifer* | F | Kampong Thom | Rainy | 12°53'15.2"N; 104°36'18.8"E | NSP09 | September-2020 |
| TS71 | *Neodartus* sp*.* | M | Kampong Thom | Rainy | 12°53'15.2"N; 104°36'18.8"E | NSP09 | September-2020 |
| TS72 | *Neodartus* sp*.* | M | Kampong Thom | Rainy | 12°53'15.2"N; 104°36'18.8"E | NSP09 | September-2020 |
| TS73 | *Neodartus* sp*.* | F | Kampong Thom | Rainy | 12°53'15.2"N; 104°36'18.8"E | NSP09 | September-2020 |
| TS74 | *Neodartus* sp*.* | F | Kampong Thom | Rainy | 12°53'15.2"N; 104°36'18.8"E | NSP09 | September-2020 |
| TS75 | *Cofana spectra* | M | Kampong Thom | Rainy | 12°53'15.2"N; 104°36'18.8"E | NSP09 | September-2020 |
| TS76 | *Cofana spectra* | F | Kampong Thom | Rainy | 12°53'15.2"N; 104°36'18.8"E | NSP09 | September-2020 |
| TS77 | *Cofana spectra* | F | Kampong Thom | Rainy | 12°53'15.2"N; 104°36'18.8"E | NSP09 | September-2020 |
| TS78 | *Cofana spectra* | F | Kampong Thom | Rainy | 12°53'15.2"N; 104°36'18.8"E | NSP09 | September-2020 |
| TS79 | *Stirellus* sp1*.* | M | Kampong Thom | Rainy | 12°53'15.2"N; 104°36'18.8"E | NSP09 | September-2020 |
| TS80 | *Stirellus* sp1 | M | Kampong Thom | Rainy | 12°53'15.2"N; 104°36'18.8"E | NSP09 | September-2020 |
| TS81 | *Stirellus* sp1 | F | Kampong Thom | Rainy | 12°53'15.2"N; 104°36'18.8"E | NSP09 | September-2020 |
| TS82 | *Stirellus* sp1 | F | Kampong Thom | Rainy | 12°53'15.2"N; 104°36'18.8"E | NSP09 | September-2020 |
| TS83 | *Stirellus* sp2. | M | Kampong Thom | Rainy | 12°53'15.2"N; 104°36'18.8"E | NSP09 | September-2020 |
| TS84 | *Stirellus* sp2. | M | Kampong Thom | Rainy | 12°53'15.2"N; 104°36'18.8"E | NSP09 | September-2020 |
| TS85 | *Stirellus* sp2. | F | Kampong Thom | Rainy | 12°53'15.2"N; 104°36'18.8"E | NSP09 | September-2020 |
| TS86 | *Stirellus* sp2. | F | Kampong Thom | Rainy | 12°53'15.2"N; 104°36'18.8"E | NSP09 | September-2020 |
| TS87 | *Batracomorphus angustatus* | F | Kampong Thom | Rainy | 12°53'15.2"N; 104°36'18.8"E | NSP09 | September-2020 |
| TS88 | *Batracomorphus angustatus* | F | Kampong Thom | Rainy | 12°53'15.2"N; 104°36'18.8"E | NSP09 | September-2020 |
| TS89 | *Stirellus capitatus* | M | Kampong Thom | Rainy | 12°53'15.2"N; 104°36'18.8"E | NSP09 | September-2020 |
| TS90 | *Stirellus capitatus* | M | Kampong Thom | Rainy | 12°53'15.2"N; 104°36'18.8"E | NSP09 | September-2020 |
| TS91 | *Stirellus capitatus* | F | Kampong Thom | Rainy | 12°53'15.2"N; 104°36'18.8"E | NSP09 | September-2020 |
| TS92 | *Stirellus capitatus* | F | Kampong Thom | Rainy | 12°53'15.2"N; 104°36'18.8"E | NSP09 | September-2020 |
| TS93 | *Hishimonus* sp*.* | F | Kampong Thom | Rainy | 12°53'15.2"N; 104°36'18.8"E | NSP09 | September-2020 |
| TS94 | *Hishimonus* sp*.* | F | Kampong Thom | Rainy | 12°53'15.2"N; 104°36'18.8"E | NSP09 | September-2020 |
| TS95 | *Goniagnathus punctifer* | M | Battambang | Dry | 13°13'9.87"N; 103° 6'5.43"E | NSP05 | January-2020 |
| TS96 | *Goniagnathus punctifer* | M | Battambang | Dry | 13°13'9.87"N; 103° 6'5.43"E | NSP05 | January-2020 |
| TS97 | *Goniagnathus punctifer* | F | Battambang | Dry | 13°13'9.87"N; 103° 6'5.43"E | NSP05 | January-2020 |
| TS98 | *Neodartus* sp*.* | M | Battambang | Dry | 13°13'9.87"N; 103° 6'5.43"E | NSP05 | January-2020 |
| TS99 | *Neodartus* sp*.* | M | Battambang | Dry | 13°13'9.87"N; 103° 6'5.43"E | NSP05 | January-2020 |
| TS100 | *Neodartus* sp*.* | F | Battambang | Dry | 13°13'9.87"N; 103° 6'5.43"E | NSP05 | January-2020 |
| TS101 | *Neodartus* sp*.* | F | Battambang | Dry | 13°13'9.87"N; 103° 6'5.43"E | NSP05 | January-2020 |
| TS102 | *Cofana spectra* | M | Battambang | Dry | 13°13'9.87"N; 103° 6'5.43"E | NSP05 | January-2020 |
| TS103 | *Cofana spectra* | F | Battambang | Dry | 13°13'9.87"N; 103° 6'5.43"E | NSP05 | January-2020 |
| TS104 | *Cofana spectra* | F | Battambang | Dry | 13°13'9.87"N; 103° 6'5.43"E | NSP05 | January-2020 |
| TS105 | *Cofana spectra* | F | Battambang | Dry | 13°13'9.87"N; 103° 6'5.43"E | NSP05 | January-2020 |
| TS106 | *Hecalus arcuatus* | M | Battambang | Dry | 13°13'9.87"N; 103° 6'5.43"E | NSP05 | January-2020 |
| TS107 | *Hecalus arcuatus* | M | Battambang | Dry | 13°13'9.87"N; 103° 6'5.43"E | NSP05 | January-2020 |
| TS108 | *Hecalus arcuatus* | F | Battambang | Dry | 13°13'9.87"N; 103° 6'5.43"E | NSP05 | January-2020 |
| TS109 | *Hecalus arcuatus* | F | Battambang | Dry | 13°13'9.87"N; 103° 6'5.43"E | NSP05 | January-2020 |
| TS110 | *Deltocephalini* sp*.* | M | Battambang | Dry | 13°13'9.87"N; 103° 6'5.43"E | NSP05 | January-2020 |
| TS111 | *Deltocephalini* sp*.* | M | Battambang | Dry | 13°13'9.87"N; 103° 6'5.43"E | NSP05 | January-2020 |
| TS112 | *Deltocephalini* sp*.* | F | Battambang | Dry | 13°13'9.87"N; 103° 6'5.43"E | NSP05 | January-2020 |
| TS113 | *Deltocephalini* sp*.* | F | Battambang | Dry | 13°13'9.87"N; 103° 6'5.43"E | NSP05 | January-2020 |
| TS114 | *Balclutha* sp*.* | M | Battambang | Dry | 13°13'9.87"N; 103° 6'5.43"E | NSP05 | January-2020 |
| TS115 | *Balclutha* sp*.* | M | Battambang | Dry | 13°13'9.87"N; 103° 6'5.43"E | NSP05 | January-2020 |
| TS116 | *Balclutha* sp*.* | F | Battambang | Dry | 13°13'9.87"N; 103° 6'5.43"E | NSP05 | January-2020 |
| TS117 | *Balclutha* sp*.* | F | Battambang | Dry | 13°13'9.87"N; 103° 6'5.43"E | NSP05 | January-2020 |
| TS118 | *Stirellus* sp2. | M | Battambang | Dry | 13°13'9.87"N; 103° 6'5.43"E | NSP05 | January-2020 |
| TS119 | *Stirellus* sp2. | M | Battambang | Dry | 13°13'9.87"N; 103° 6'5.43"E | NSP05 | January-2020 |
| TS120 | *Stirellus* sp2. | F | Battambang | Dry | 13°13'9.87"N; 103° 6'5.43"E | NSP05 | January-2020 |
| TS121 | *Stirellus* sp2. | F | Battambang | Dry | 13°13'9.87"N; 103° 6'5.43"E | NSP05 | January-2020 |
| TS122 | *Maiestas* sp*.* | M | Battambang | Dry | 13°13'9.87"N; 103° 6'5.43"E | NSP05 | January-2020 |
| TS123 | *Maiestas* sp*.* | F | Battambang | Dry | 13°13'9.87"N; 103° 6'5.43"E | NSP05 | January-2020 |
| TS124 | *Stirellus capitatus* | M | Battambang | Dry | 13°13'9.87"N; 103° 6'5.43"E | NSP05 | January-2020 |
| TS125 | *Stirellus capitatus* | M | Battambang | Dry | 13°13'9.87"N; 103° 6'5.43"E | NSP05 | January-2020 |
| TS126 | *Stirellus capitatus* | F | Battambang | Dry | 13°13'9.87"N; 103° 6'5.43"E | NSP05 | January-2020 |

**Table S2**: Read count tracking of sequence reads by sample of the Illumina Miseq (V3-V4) amplicons

| **Sam.ID** | **Host Species** | **dada2 input** | **filtered** | **dada_F** | **dada_R** | **Merged** | **Nonchim** | **Final_reads (%)** |
| --- | --- | --- | --- | --- | --- | --- | --- | --- |
| TS01 | *Nephotettix virescens* | 12292 | 12285 | 12244 | 12259 | 12163 | 11043 | 89.80 |
| TS02 | *Nephotettix virescens* | 1359 | 1358 | 1354 | 1355 | 1342 | 1184 | 87.10 |
| TS03 | *Nephotettix virescens* | 12532 | 12519 | 12196 | 12269 | 11521 | 9370 | 74.80 |
| TS04 | *Nephotettix virescens* | 6183 | 6177 | 6174 | 6170 | 6142 | 5305 | 85.80 |
| TS05 | *Nephotettix virescens* | 10625 | 10616 | 10586 | 10593 | 10517 | 9588 | 90.20 |
| TS06 | *Nephotettix virescens* | 11701 | 11686 | 11648 | 11663 | 11585 | 9876 | 84.40 |
| TS07 | *Nephotettix virescens* | 8768 | 8759 | 8742 | 8740 | 8688 | 7713 | 88.00 |
| TS08 | *Nephotettix virescens* | 3523 | 3521 | 3517 | 3520 | 3310 | 3073 | 87.20 |
| TS09 | *Nephotettix nigropictus* | 3312 | 3309 | 3275 | 3278 | 3229 | 3034 | 91.60 |
| TS10 | *Nephotettix nigropictus* | 727 | 726 | 725 | 725 | 717 | 696 | 95.70 |
| TS11 | *Nephotettix virescens* | 6991 | 6987 | 6972 | 6981 | 6923 | 6237 | 89.20 |
| TS12 | *Nephotettix virescens* | 1362 | 1362 | 1359 | 1360 | 1349 | 1249 | 91.70 |
| TS13 | *Nephotettix virescens* | 4764 | 4760 | 4749 | 4751 | 4712 | 4302 | 90.30 |
| TS14 | *Nephotettix virescens* | 15292 | 15275 | 15251 | 15261 | 15162 | 13541 | 88.50 |
| TS15 | *Nephotettix virescens* | 11267 | 11263 | 11246 | 11255 | 11175 | 10005 | 88.80 |
| TS16 | *Nephotettix virescens* | 29845 | 29818 | 29732 | 29756 | 29520 | 27236 | 91.30 |
| TS17 | *Nephotettix virescens* | 17025 | 17003 | 16924 | 16942 | 16799 | 14258 | 83.70 |
| TS18 | *Nephotettix virescens* | 49266 | 49225 | 49134 | 49151 | 48750 | 43998 | 89.30 |
| TS19 | *Nephotettix virescens* | 54030 | 53984 | 53895 | 53932 | 53536 | 48433 | 89.60 |
| TS20 | *Nephotettix virescens* | 1876 | 1875 | 1874 | 1871 | 1864 | 1817 | 96.90 |
| TS21 | *Nephotettix virescens* | 12208 | 12191 | 12159 | 12169 | 12069 | 11226 | 92.00 |
| TS22 | *Nephotettix virescens* | 25317 | 25286 | 25242 | 25243 | 25079 | 22575 | 89.20 |
| TS23 | *Nephotettix virescens* | 21754 | 21727 | 21682 | 21703 | 21540 | 19701 | 90.60 |
| TS24 | *Nephotettix virescens* | 28329 | 28301 | 28199 | 28221 | 27975 | 25566 | 90.20 |
| TS25 | *Nephotettix virescens* | 4492 | 4491 | 4476 | 4480 | 3895 | 3470 | 77.20 |
| TS26 | *Nephotettix virescens* | 5734 | 5728 | 5710 | 5716 | 5674 | 5001 | 87.20 |
| TS27 | *Nephotettix virescens* | 2205 | 2201 | 2195 | 2196 | 2173 | 1951 | 88.50 |
| TS28 | *Nephotettix virescens* | 11792 | 11778 | 11758 | 11766 | 11411 | 10389 | 88.10 |
| TS29 | *Nephotettix virescens* | 6053 | 6047 | 6037 | 6041 | 5990 | 5223 | 86.30 |
| TS30 | *Nephotettix virescens* | 10912 | 10902 | 10888 | 10891 | 10803 | 9854 | 90.30 |
| TS31 | *Nephotettix virescens* | 21120 | 21098 | 21068 | 21077 | 20923 | 18796 | 89.00 |
| TS32 | *Nephotettix virescens* | 11743 | 11736 | 11717 | 11720 | 11642 | 10752 | 91.60 |
| TS33 | *Nephotettix virescens* | 7825 | 7815 | 7784 | 7789 | 7728 | 7031 | 89.90 |
| TS34 | *Nephotettix virescens* | 5983 | 5977 | 5967 | 5968 | 5934 | 5302 | 88.60 |
| TS35 | *Nephotettix virescens* | 32721 | 32684 | 32625 | 32635 | 32395 | 28810 | 88.00 |
| TS36 | *Nephotettix nigropictus* | 4383 | 4380 | 4363 | 4371 | 3947 | 3893 | 88.80 |
| TS37 | *Nephotettix nigropictus* | 22526 | 22499 | 22396 | 22412 | 22125 | 21161 | 93.90 |
| TS38 | *Nephotettix nigropictus* | 3264 | 3258 | 3240 | 3249 | 3217 | 3076 | 94.20 |
| TS39 | *Nephotettix virescens* | 5322 | 5317 | 5305 | 5311 | 5021 | 4370 | 82.10 |
| TS40 | *Exitiainus indicus* | 6273 | 6267 | 6211 | 6233 | 6075 | 5706 | 91.00 |
| TS41 | *Exitiainus indicus* | 4765 | 4759 | 4675 | 4693 | 4585 | 4444 | 93.30 |
| TS42 | *Exitiainus indicus* | 15330 | 15313 | 15254 | 15268 | 15146 | 14113 | 92.10 |
| TS43 | *Maiestas dorsalis* | 2496 | 2496 | 2480 | 2485 | 2255 | 2065 | 82.70 |
| TS44 | *Maiestas dorsalis* | 10849 | 10839 | 10793 | 10805 | 9944 | 8915 | 82.20 |
| TS45 | *Maiestas dorsalis* | 12764 | 12752 | 12616 | 12613 | 11795 | 11109 | 87.00 |
| TS46 | *Maiestas dorsalis* | 2919 | 2918 | 2910 | 2913 | 2642 | 2345 | 80.30 |
| TS47 | *Exitiainus indicus* | 16052 | 16030 | 15985 | 16001 | 15793 | 14005 | 87.20 |
| TS48 | *Exitiainus indicus* | 3577 | 3575 | 3553 | 3568 | 3496 | 3243 | 90.70 |
| TS49 | *Exitiainus indicus* | 5371 | 5367 | 5353 | 5364 | 5261 | 4978 | 92.70 |
| TS50 | *Exitiainus indicus* | 14189 | 14177 | 14079 | 14124 | 13797 | 12836 | 90.50 |
| TS51 | *Maiestas dorsalis* | 4873 | 4868 | 4824 | 4842 | 4445 | 3982 | 81.70 |
| TS52 | *Maiestas dorsalis* | 2809 | 2809 | 2757 | 2763 | 2523 | 2347 | 83.60 |
| TS53 | *Maiestas dorsalis* | 47452 | 47416 | 47265 | 47294 | 44320 | 42481 | 89.50 |
| TS54 | *Maiestas dorsalis* | 5822 | 5819 | 5808 | 5810 | 5437 | 4976 | 85.50 |
| TS55 | *Exitiainus indicus* | 7170 | 7165 | 7146 | 7147 | 7105 | 6728 | 93.80 |
| TS56 | *Exitiainus indicus* | 13989 | 13978 | 13916 | 13937 | 13812 | 13533 | 96.70 |
| TS57 | *Exitiainus indicus* | 26034 | 26009 | 25936 | 25944 | 25763 | 24860 | 95.50 |
| TS58 | *Exitiainus indicus* | 11559 | 11549 | 11518 | 11524 | 11442 | 11019 | 95.30 |
| TS59 | *Maiestas dorsalis* | 2800 | 2797 | 2736 | 2751 | 2543 | 2309 | 82.50 |
| TS60 | *Maiestas dorsalis* | 1083 | 1082 | 1074 | 1076 | 986 | 902 | 83.30 |
| TS61 | *Maiestas dorsalis* | 19114 | 19096 | 19048 | 19045 | 17556 | 16479 | 86.20 |
| TS62 | *Maiestas dorsalis* | 4188 | 4183 | 4171 | 4169 | 3849 | 3627 | 86.60 |
| TS63 | *Hecalus* sp*.* | 9929 | 9914 | 9858 | 9872 | 9568 | 9204 | 92.70 |
| TS64 | *Hecalus* sp*.* | 19054 | 19033 | 18938 | 18965 | 18538 | 17646 | 92.60 |
| TS65 | *Hecalus* sp*.* | 10406 | 10400 | 10363 | 10380 | 10075 | 9818 | 94.30 |
| TS66 | *Hecalus* sp*.* | 11491 | 11478 | 11447 | 11459 | 11114 | 10693 | 93.10 |
| TS67 | *Goniagnathus punctifer* | 9108 | 9101 | 9059 | 9069 | 9042 | 8687 | 95.40 |
| TS68 | *Goniagnathus punctifer* | 25283 | 25256 | 25184 | 25196 | 25128 | 23905 | 94.50 |
| TS69 | *Goniagnathus punctifer* | 17966 | 17949 | 17904 | 17919 | 17860 | 17113 | 95.30 |
| TS70 | *Goniagnathus punctifer* | 1410 | 1409 | 1409 | 1408 | 1407 | 1358 | 96.30 |
| TS71 | *Neodartus* sp*.* | 9994 | 9981 | 9943 | 9949 | 9897 | 9195 | 92.00 |
| TS72 | *Neodartus* sp*.* | 20805 | 20785 | 20745 | 20738 | 20640 | 19180 | 92.20 |
| TS73 | *Neodartus* sp*.* | 11743 | 11736 | 11713 | 11714 | 11671 | 10911 | 92.90 |
| TS74 | *Neodartus* sp*.* | 1958 | 1955 | 1950 | 1952 | 1938 | 1739 | 88.80 |
| TS75 | *Cofana spectra* | 26540 | 26515 | 26453 | 26452 | 26401 | 24061 | 90.70 |
| TS76 | *Cofana spectra* | 10134 | 10126 | 10104 | 10104 | 10029 | 9217 | 91.00 |
| TS77 | *Cofana spectra* | 4679 | 4677 | 4667 | 4662 | 4629 | 4222 | 90.20 |
| TS78 | *Cofana spectra* | 16746 | 16730 | 16697 | 16697 | 16528 | 15583 | 93.10 |
| TS79 | *Stirellus* sp1*.* | 1028 | 1028 | 1016 | 1018 | 1004 | 930 | 90.50 |
| TS80 | *Stirellus* sp1 | 351 | 351 | 344 | 343 | 337 | 316 | 90.00 |
| TS81 | *Stirellus* sp1 | 22672 | 22655 | 22609 | 22626 | 22432 | 20759 | 91.60 |
| TS82 | *Stirellus* sp1 | 10616 | 10608 | 10550 | 10548 | 10461 | 9708 | 91.40 |
| TS83 | *Stirellus* sp2. | 3510 | 3509 | 3482 | 3481 | 3448 | 3194 | 91.00 |
| TS84 | *Stirellus* sp2. | 4447 | 4440 | 4407 | 4410 | 4376 | 4107 | 92.40 |
| TS85 | *Stirellus* sp2. | 11545 | 11538 | 11494 | 11493 | 11412 | 10887 | 94.30 |
| TS86 | *Stirellus* sp2. | 4246 | 4242 | 4236 | 4239 | 4199 | 3939 | 92.80 |
| TS87 | *Batracomorphus angustatus* | 16341 | 16321 | 16227 | 16248 | 16148 | 14855 | 90.90 |
| TS88 | *Batracomorphus angustatus* | 4793 | 4786 | 4749 | 4759 | 4731 | 4374 | 91.30 |
| TS89 | *Stirellus capitatus* | 11885 | 11875 | 11822 | 11823 | 10602 | 9292 | 78.20 |
| TS90 | *Stirellus capitatus* | 7305 | 7298 | 7260 | 7274 | 6995 | 5855 | 80.20 |
| TS91 | *Stirellus capitatus* | 9222 | 9213 | 9191 | 9202 | 8063 | 7259 | 78.70 |
| TS92 | *Stirellus capitatus* | 5682 | 5677 | 5664 | 5666 | 5059 | 4386 | 77.20 |
| TS93 | *Hishimonus* sp*.* | 15016 | 14997 | 14954 | 14957 | 14789 | 13844 | 92.20 |
| TS94 | *Hishimonus* sp*.* | 12019 | 12009 | 11948 | 11952 | 11811 | 10998 | 91.50 |
| TS95 | *Goniagnathus punctifer* | 19097 | 19074 | 19055 | 19047 | 19006 | 18296 | 95.80 |
| TS96 | *Goniagnathus punctifer* | 1997 | 1994 | 1985 | 1984 | 1977 | 1808 | 90.50 |
| TS97 | *Goniagnathus punctifer* | 11891 | 11879 | 11856 | 11837 | 11813 | 11205 | 94.20 |
| TS98 | *Neodartus* sp*.* | 6834 | 6829 | 6683 | 6692 | 6536 | 5540 | 81.10 |
| TS99 | *Neodartus* sp*.* | 4586 | 4581 | 4557 | 4561 | 4527 | 4158 | 90.70 |
| TS100 | *Neodartus* sp*.* | 1808 | 1808 | 1807 | 1808 | 1799 | 1696 | 93.80 |
| TS101 | *Neodartus* sp*.* | 17919 | 17903 | 17872 | 17866 | 17777 | 16985 | 94.80 |
| TS102 | *Cofana spectra* | 1751 | 1747 | 1744 | 1744 | 1733 | 1521 | 86.90 |
| TS103 | *Cofana spectra* | 10209 | 10199 | 10167 | 10175 | 10140 | 9748 | 95.50 |
| TS104 | *Cofana spectra* | 6514 | 6508 | 6491 | 6494 | 6442 | 6116 | 93.90 |
| TS105 | *Cofana spectra* | 9359 | 9353 | 9318 | 9331 | 9295 | 8765 | 93.70 |
| TS106 | *Hecalus arcuatus* | 15743 | 15718 | 15651 | 15659 | 15307 | 14287 | 90.80 |
| TS107 | *Hecalus arcuatus* | 20499 | 20480 | 20397 | 20399 | 19962 | 18608 | 90.80 |
| TS108 | *Hecalus arcuatus* | 20220 | 20205 | 20147 | 20133 | 19658 | 18874 | 93.30 |
| TS109 | *Hecalus arcuatus* | 9827 | 9817 | 9796 | 9803 | 9632 | 9156 | 93.20 |
| TS110 | *Deltocephalini* sp*.* | 1496 | 1495 | 1490 | 1495 | 1486 | 1374 | 91.80 |
| TS111 | *Deltocephalini* sp*.* | 8206 | 8197 | 8179 | 8189 | 8159 | 7701 | 93.80 |
| TS112 | *Deltocephalini* sp*.* | 3397 | 3394 | 3389 | 3393 | 3376 | 3221 | 94.80 |
| TS113 | *Deltocephalini* sp*.* | 15500 | 15486 | 15436 | 15468 | 15412 | 14668 | 94.60 |
| TS114 | *Balclutha* sp*.* | 7431 | 7427 | 7381 | 7382 | 7237 | 6444 | 86.70 |
| TS115 | *Balclutha* sp*.* | 11554 | 11540 | 11475 | 11472 | 11221 | 9939 | 86.00 |
| TS116 | *Balclutha* sp*.* | 8085 | 8076 | 8039 | 8033 | 7834 | 7041 | 87.10 |
| TS117 | *Balclutha* sp*.* | 11436 | 11427 | 11399 | 11384 | 11081 | 10287 | 90.00 |
| TS118 | *Stirellus* sp2. | 26394 | 26363 | 26314 | 26320 | 26206 | 24851 | 94.20 |
| TS119 | *Stirellus* sp2. | 31759 | 31740 | 31628 | 31657 | 31466 | 29774 | 93.70 |
| TS120 | *Stirellus* sp2. | 1708 | 1706 | 1702 | 1703 | 1693 | 1594 | 93.30 |
| TS121 | *Stirellus* sp2. | 17079 | 17063 | 16992 | 16995 | 16895 | 15949 | 93.40 |
| TS122 | *Maiestas* sp*.* | 8012 | 8003 | 7945 | 7953 | 7594 | 6789 | 84.70 |
| TS123 | *Maiestas* sp*.* | 18390 | 18373 | 18298 | 18320 | 17596 | 16426 | 89.30 |
| TS124 | *Stirellus capitatus* | 32008 | 31973 | 31927 | 31921 | 30204 | 24595 | 76.80 |
| TS125 | *Stirellus capitatus* | 44329 | 44286 | 44182 | 44187 | 40871 | 35163 | 79.30 |
| TS126 | *Stirellus capitatus* | 12485 | 12479 | 12462 | 12466 | 10699 | 9318 | 74.60 |

**Table S3**: Relative abundance (sequence reads) of bacterial communities in association with Cicadellidae insects

| **ASVs** | **Phylum** | **Class** | **Order** | **Family** | **Genus** | **ASVs Abundance** |
| --- | --- | --- | --- | --- | --- | --- |
| ASV_1 | Bacteroidota | Bacteroidia | Flavobacteriales | Blattabacteriaceae | Candidatus Karelsulcia | 180228 |
| ASV_2 | Proteobacteria | Gammaproteobacteria | Burkholderiales | Oxalobacteraceae | Candidatus Nasuia | 132862 |
| ASV_3 | Bacteroidota | Bacteroidia | Flavobacteriales | Blattabacteriaceae | Candidatus Karelsulcia | 95224 |
| ASV_4 | Bacteroidota | Bacteroidia | Flavobacteriales | Blattabacteriaceae | Candidatus Karelsulcia | 88655 |
| ASV_5 | Bacteroidota | Bacteroidia | Flavobacteriales | Blattabacteriaceae | Candidatus Karelsulcia | 69291 |
| ASV_6 | Bacteroidota | Bacteroidia | Flavobacteriales | Blattabacteriaceae | Candidatus Karelsulcia | 59078 |
| ASV_8 | Proteobacteria | Gammaproteobacteria | Diplorickettsiales | Diplorickettsiaceae | Diplorickettsia | 51432 |
| ASV_9 | Bacteroidota | Bacteroidia | Flavobacteriales | Blattabacteriaceae | Candidatus Karelsulcia | 49892 |
| ASV_11 | Proteobacteria | Alphaproteobacteria | Rickettsiales | Anaplasmataceae | Wolbachia | 38635 |
| ASV_12 | Proteobacteria | Gammaproteobacteria | Diplorickettsiales | Diplorickettsiaceae | Diplorickettsia | 37987 |
| ASV_13 | Bacteroidota | Bacteroidia | Flavobacteriales | Blattabacteriaceae | Candidatus Karelsulcia | 36543 |
| ASV_14 | Proteobacteria | Alphaproteobacteria | Rickettsiales | Anaplasmataceae | Wolbachia | 36472 |
| ASV_16 | Bacteroidota | Bacteroidia | Flavobacteriales | Blattabacteriaceae | Candidatus Karelsulcia | 24754 |
| ASV_18 | Proteobacteria | Gammaproteobacteria | Burkholderiales | Oxalobacteraceae | Candidatus Nasuia | 23672 |
| ASV_19 | Proteobacteria | Gammaproteobacteria | Burkholderiales | Oxalobacteraceae | Candidatus Nasuia | 21259 |
| ASV_22 | Bacteroidota | Bacteroidia | Flavobacteriales | Blattabacteriaceae | Candidatus Karelsulcia | 20263 |
| ASV_23 | Bacteroidota | Bacteroidia | Flavobacteriales | Blattabacteriaceae | Candidatus Karelsulcia | 20091 |
| ASV_24 | Proteobacteria | Gammaproteobacteria | unclassified_Gammaproteobacteria | NA | NA | 16688 |
| ASV_25 | Bacteroidota | Bacteroidia | Flavobacteriales | Blattabacteriaceae | Candidatus Karelsulcia | 14437 |
| ASV_26 | Proteobacteria | Gammaproteobacteria | unclassified_Gammaproteobacteria | NA | NA | 14108 |
| ASV_27 | Proteobacteria | Gammaproteobacteria | Burkholderiales | Oxalobacteraceae | Candidatus Nasuia | 13377 |
| ASV_28 | Bacteroidota | Bacteroidia | Flavobacteriales | Blattabacteriaceae | Candidatus Karelsulcia | 12187 |
| ASV_29 | Bacteroidota | Bacteroidia | Flavobacteriales | Blattabacteriaceae | Candidatus Karelsulcia | 11639 |
| ASV_30 | Proteobacteria | Gammaproteobacteria | Burkholderiales | Oxalobacteraceae | Candidatus Nasuia | 8851 |
| ASV_33 | Proteobacteria | Alphaproteobacteria | Rickettsiales | Anaplasmataceae | Wolbachia | 6564 |
| ASV_35 | Proteobacteria | Gammaproteobacteria | Enterobacterales | Morganellaceae | Arsenophonus | 6166 |
| ASV_38 | Firmicutes | Bacilli | Lactobacillales | Streptococcaceae | Lactococcus | 3741 |
| ASV_39 | Proteobacteria | Gammaproteobacteria | Enterobacterales | Pectobacteriaceae | Pectobacterium | 3478 |
| ASV_41 | Proteobacteria | Gammaproteobacteria | Diplorickettsiales | Diplorickettsiaceae | Diplorickettsia | 3118 |
| ASV_42 | Proteobacteria | Gammaproteobacteria | Diplorickettsiales | Diplorickettsiaceae | Diplorickettsia | 2636 |
| ASV_43 | Proteobacteria | Alphaproteobacteria | Rickettsiales | Anaplasmataceae | Wolbachia | 2343 |
| ASV_44 | Bacteroidota | Bacteroidia | Flavobacteriales | Blattabacteriaceae | Candidatus Karelsulcia | 1930 |
| ASV_45 | Proteobacteria | Gammaproteobacteria | Diplorickettsiales | Diplorickettsiaceae | Diplorickettsia | 1795 |
| ASV_46 | Proteobacteria | Alphaproteobacteria | Rickettsiales | Rickettsiaceae | Rickettsia | 1625 |
| ASV_47 | Proteobacteria | Gammaproteobacteria | Enterobacterales | Morganellaceae | Arsenophonus | 1578 |
| ASV_48 | Proteobacteria | Gammaproteobacteria | Enterobacterales | Pectobacteriaceae | Sodalis | 1185 |
| ASV_49 | Proteobacteria | Gammaproteobacteria | Burkholderiales | Oxalobacteraceae | Candidatus Nasuia | 1114 |
| ASV_50 | Proteobacteria | Gammaproteobacteria | Enterobacterales | Morganellaceae | unclassified_Morganellaceae | 1056 |
| ASV_51 | Proteobacteria | Gammaproteobacteria | Diplorickettsiales | Diplorickettsiaceae | Diplorickettsia | 1048 |
| ASV_52 | Proteobacteria | Gammaproteobacteria | Enterobacterales | Pectobacteriaceae | Sodalis | 849 |
| ASV_53 | Proteobacteria | Gammaproteobacteria | Enterobacterales | Pectobacteriaceae | Sodalis | 754 |
| ASV_54 | Bacteroidota | Bacteroidia | Flavobacteriales | Blattabacteriaceae | Candidatus Karelsulcia | 750 |
| ASV_55 | Proteobacteria | Gammaproteobacteria | Diplorickettsiales | Diplorickettsiaceae | Diplorickettsia | 713 |
| ASV_56 | Proteobacteria | Gammaproteobacteria | Enterobacterales | Pectobacteriaceae | Sodalis | 668 |
| ASV_57 | Proteobacteria | Gammaproteobacteria | Enterobacterales | Pectobacteriaceae | Sodalis | 648 |
| ASV_58 | Bacteroidota | Bacteroidia | Flavobacteriales | Blattabacteriaceae | Candidatus Karelsulcia | 634 |
| ASV_59 | Proteobacteria | Alphaproteobacteria | Rickettsiales | Fokiniaceae | Candidatus Lariskella | 597 |
| ASV_60 | Proteobacteria | Gammaproteobacteria | Enterobacterales | Pectobacteriaceae | Sodalis | 589 |
| ASV_61 | Proteobacteria | Gammaproteobacteria | Burkholderiales | Oxalobacteraceae | Candidatus Nasuia | 519 |
| ASV_63 | Proteobacteria | Alphaproteobacteria | Rickettsiales | Fokiniaceae | Candidatus Lariskella | 420 |
| ASV_64 | Proteobacteria | Gammaproteobacteria | Enterobacterales | Pectobacteriaceae | Sodalis | 416 |
| ASV_65 | Bacteroidota | Bacteroidia | Flavobacteriales | Blattabacteriaceae | Candidatus Karelsulcia | 362 |
| ASV_68 | Bacteroidota | Bacteroidia | Flavobacteriales | Blattabacteriaceae | Candidatus Karelsulcia | 313 |
| ASV_70 | Proteobacteria | Gammaproteobacteria | Enterobacterales | Pectobacteriaceae | Sodalis | 288 |
| ASV_71 | Proteobacteria | Gammaproteobacteria | Enterobacterales | Erwiniaceae | Pantoea | 277 |
| ASV_72 | Proteobacteria | Gammaproteobacteria | Enterobacterales | Pectobacteriaceae | Sodalis | 262 |
| ASV_73 | Proteobacteria | Gammaproteobacteria | Enterobacterales | Pectobacteriaceae | Sodalis | 257 |
| ASV_74 | Proteobacteria | Gammaproteobacteria | Enterobacterales | unclassified_Enterobacterales | NA | 247 |
| ASV_75 | Proteobacteria | Gammaproteobacteria | Enterobacterales | Pectobacteriaceae | Sodalis | 140 |
| ASV_76 | Proteobacteria | Gammaproteobacteria | Enterobacterales | Pectobacteriaceae | Sodalis | 116 |
| ASV_77 | Actinobacteriota | Actinobacteria | Micrococcales | Microbacteriaceae | unclassified_Microbacteriaceae | 101 |
| ASV_78 | Proteobacteria | Alphaproteobacteria | Rhizobiales | Beijerinckiaceae | Methylobacterium-Methylorubrum | 99 |
| ASV_80 | Bacteroidota | Bacteroidia | Flavobacteriales | Flavobacteriaceae | Flavobacterium | 69 |
| ASV_84 | Firmicutes | Bacilli | Bacillales | Bacillaceae | Bacillus | 45 |
| ASV_85 | Proteobacteria | Gammaproteobacteria | Diplorickettsiales | Diplorickettsiaceae | Diplorickettsia | 44 |
| ASV_86 | Proteobacteria | Alphaproteobacteria | Rhizobiales | Rhizobiaceae | Aureimonas | 44 |
| ASV_87 | Bacteroidota | Bacteroidia | Flavobacteriales | Blattabacteriaceae | Candidatus Karelsulcia | 41 |
| ASV_88 | Proteobacteria | Alphaproteobacteria | Sphingomonadales | Sphingomonadaceae | Sphingomonas | 34 |
| ASV_89 | Proteobacteria | Gammaproteobacteria | Pseudomonadales | Pseudomonadaceae | Pseudomonas | 32 |
| ASV_90 | Proteobacteria | Alphaproteobacteria | Rhizobiales | Xanthobacteraceae | unclassified_Xanthobacteraceae | 31 |
| ASV_91 | Proteobacteria | Gammaproteobacteria | Burkholderiales | Comamonadaceae | unclassified_Comamonadaceae | 30 |
| ASV_92 | Proteobacteria | Alphaproteobacteria | Acetobacterales | Acetobacteraceae | unclassified_Acetobacteraceae | 27 |
| ASV_93 | Proteobacteria | Gammaproteobacteria | unclassified_Gammaproteobacteria | NA | NA | 24 |
| ASV_94 | Proteobacteria | Gammaproteobacteria | Burkholderiales | unclassified_Burkholderiales | NA | 22 |
| ASV_95 | Bacteroidota | Bacteroidia | Flavobacteriales | Blattabacteriaceae | Candidatus Karelsulcia | 22 |
| ASV_96 | Proteobacteria | Alphaproteobacteria | Rhizobiales | Rhizobiaceae | Allorhizobium-Neorhizobium-Pararhizobium-Rhizobium | 20 |
| ASV_99 | Proteobacteria | Alphaproteobacteria | Rhizobiales | Rhizobiales Incertae Sedis | Phreatobacter | 18 |
| ASV_100 | Actinobacteriota | Actinobacteria | Corynebacteriales | Nocardiaceae | Williamsia | 17 |
| ASV_101 | Bacteroidota | Bacteroidia | Flavobacteriales | Blattabacteriaceae | Candidatus Karelsulcia | 17 |
| ASV_102 | Actinobacteriota | Actinobacteria | Pseudonocardiales | Pseudonocardiaceae | Actinomycetospora | 16 |
| ASV_103 | Bacteroidota | Bacteroidia | Flavobacteriales | Blattabacteriaceae | Candidatus Karelsulcia | 16 |
| ASV_106 | Firmicutes | Bacilli | Exiguobacterales | Exiguobacteraceae | Exiguobacterium | 14 |
| ASV_107 | Bacteroidota | Bacteroidia | Flavobacteriales | Blattabacteriaceae | Candidatus Karelsulcia | 14 |
| ASV_108 | Bacteroidota | Bacteroidia | Chitinophagales | Chitinophagaceae | Asinibacterium | 13 |
| ASV_110 | Firmicutes | Bacilli | Lactobacillales | Streptococcaceae | Streptococcus | 11 |
| ASV_111 | Bacteroidota | Bacteroidia | Flavobacteriales | Blattabacteriaceae | Candidatus Karelsulcia | 11 |
| ASV_114 | Bacteroidota | Bacteroidia | Flavobacteriales | Blattabacteriaceae | Candidatus Karelsulcia | 10 |
| ASV_116 | Proteobacteria | Alphaproteobacteria | Sphingomonadales | Sphingomonadaceae | Altererythrobacter | 8 |
| ASV_118 | Proteobacteria | Gammaproteobacteria | Burkholderiales | Oxalobacteraceae | Candidatus Nasuia | 6 |
| ASV_119 | Bacteroidota | Bacteroidia | Flavobacteriales | Blattabacteriaceae | Candidatus Karelsulcia | 5 |
| ASV_121 | Proteobacteria | Alphaproteobacteria | Acetobacterales | Acetobacteraceae | Roseomonas | 4 |
| ASV_123 | Bacteroidota | Bacteroidia | Flavobacteriales | Blattabacteriaceae | Candidatus Karelsulcia | 3 |
| ASV_125 | Firmicutes | Clostridia | NA | Hungateiclostridiaceae | Ruminiclostridium | 2 |
| ASV_126 | Bacteroidota | Bacteroidia | Cytophagales | Hymenobacteraceae | Hymenobacter | 2 |
| ASV_127 | Bacteroidota | Bacteroidia | Flavobacteriales | Blattabacteriaceae | Candidatus Karelsulcia | 2 |
| ASV_130 | Bacteroidota | Bacteroidia | Flavobacteriales | Blattabacteriaceae | Candidatus Karelsulcia | 1 |
| ASV_131 | Bacteroidota | Bacteroidia | Flavobacteriales | Blattabacteriaceae | Candidatus Karelsulcia | 1 |
| ASV_132 | Proteobacteria | Gammaproteobacteria | Diplorickettsiales | Diplorickettsiaceae | Diplorickettsia | 1 |

**Table S4**: Analysis of Random Forest (RF) for key microbial taxa (ASVs) of male and female, *Nephotettix virescens*, based on Mean DecreaseGini

| **ASVs** | **Phylum** | **Class** | **Order** | **Family** | **Genus** | **Mean *DecreaseGini*** |
| --- | --- | --- | --- | --- | --- | --- |
| ASV_1 | Bacteroidota | Bacteroidia | Flavobacteriales | Blattabacteriaceae | Candidatus Karelsulcia | 0.344504824 |
| ASV_2 | Proteobacteria | Gammaproteobacteria | Burkholderiales | Oxalobacteraceae | Candidatus Nasuia | 0.341768919 |
| ASV_3 | Bacteroidota | Bacteroidia | Flavobacteriales | Blattabacteriaceae | Candidatus Karelsulcia | 0.133374316 |
| ASV_4 | Bacteroidota | Bacteroidia | Flavobacteriales | Blattabacteriaceae | Candidatus Karelsulcia | 0.017747619 |
| ASV_5 | Bacteroidota | Bacteroidia | Flavobacteriales | Blattabacteriaceae | Candidatus Karelsulcia | 0.035111611 |
| ASV_6 | Bacteroidota | Bacteroidia | Flavobacteriales | Blattabacteriaceae | Candidatus Karelsulcia | 0.003733333 |
| ***ASV_8*** | ***Proteobacteria*** | ***Gammaproteobacteria*** | ***Diplorickettsiales*** | ***Diplorickettsiaceae*** | ***Diplorickettsia*** | ***1.289711603*** |
| ASV_9 | Bacteroidota | Bacteroidia | Flavobacteriales | Blattabacteriaceae | Candidatus Karelsulcia | 0.083914515 |
| ASV_11 | Proteobacteria | Alphaproteobacteria | Rickettsiales | Anaplasmataceae | Wolbachia | 0.003000000 |
| ***ASV_12*** | ***Proteobacteria*** | ***Gammaproteobacteria*** | ***Diplorickettsiales*** | ***Diplorickettsiaceae*** | ***Diplorickettsia*** | ***0.727113345*** |
| ***ASV_13*** | ***Bacteroidota*** | ***Bacteroidia*** | ***Flavobacteriales*** | ***Blattabacteriaceae*** | ***Candidatus Karelsulcia*** | ***4.715535092*** |
| ASV_16 | Bacteroidota | Bacteroidia | Flavobacteriales | Blattabacteriaceae | Candidatus Karelsulcia | 0.030009524 |
| ASV_22 | Bacteroidota | Bacteroidia | Flavobacteriales | Blattabacteriaceae | Candidatus Karelsulcia | 0.014558730 |
| ASV_24 | Proteobacteria | Gammaproteobacteria | unclassified_Gammaproteobacteria | NA | NA | 0.000000000 |
| ASV_27 | Proteobacteria | Gammaproteobacteria | Burkholderiales | Oxalobacteraceae | Candidatus Nasuia | 0.067563916 |
| ASV_30 | Proteobacteria | Gammaproteobacteria | Burkholderiales | Oxalobacteraceae | Candidatus Nasuia | 0.286730508 |
| ASV_35 | Proteobacteria | Gammaproteobacteria | Enterobacterales | Morganellaceae | Arsenophonus | 0.054256939 |
| ASV_41 | Proteobacteria | Gammaproteobacteria | Diplorickettsiales | Diplorickettsiaceae | Diplorickettsia | 0.006692308 |
| ASV_42 | Proteobacteria | Gammaproteobacteria | Diplorickettsiales | Diplorickettsiaceae | Diplorickettsia | 0.441267947 |
| ASV_43 | Proteobacteria | Alphaproteobacteria | Rickettsiales | Anaplasmataceae | Wolbachia | 0.016533333 |
| ASV_44 | Bacteroidota | Bacteroidia | Flavobacteriales | Blattabacteriaceae | Candidatus Karelsulcia | 0.283460323 |
| ASV_45 | Proteobacteria | Gammaproteobacteria | Diplorickettsiales | Diplorickettsiaceae | Diplorickettsia | 0.362772244 |
| ASV_49 | Proteobacteria | Gammaproteobacteria | Burkholderiales | Oxalobacteraceae | Candidatus Nasuia | 0.420783064 |
| ASV_51 | Proteobacteria | Gammaproteobacteria | Diplorickettsiales | Diplorickettsiaceae | Diplorickettsia | 0.265689525 |
| ASV_58 | Bacteroidota | Bacteroidia | Flavobacteriales | Blattabacteriaceae | Candidatus Karelsulcia | 0.129188581 |
| ASV_61 | Proteobacteria | Gammaproteobacteria | Burkholderiales | Oxalobacteraceae | Candidatus Nasuia | 0.511657215 |
| ASV_65 | Bacteroidota | Bacteroidia | Flavobacteriales | Blattabacteriaceae | Candidatus Karelsulcia | 0.261036941 |
| ASV_68 | Bacteroidota | Bacteroidia | Flavobacteriales | Blattabacteriaceae | Candidatus Karelsulcia | 0.012514286 |
| ASV_71 | Proteobacteria | Gammaproteobacteria | Enterobacterales | Erwiniaceae | Pantoea | 0.025336752 |
| ASV_74 | Proteobacteria | Gammaproteobacteria | Enterobacterales | unclassified_Enterobacterales | NA | 0.008666667 |
| ASV_77 | Actinobacteriota | Actinobacteria | Micrococcales | Microbacteriaceae | unclassified_Microbacteriaceae | 0.020988562 |
| ASV_78 | Proteobacteria | Alphaproteobacteria | Rhizobiales | Beijerinckiaceae | Methylobacterium-Methylorubrum | 0.241492740 |
| ASV_80 | Bacteroidota | Bacteroidia | Flavobacteriales | Flavobacteriaceae | Flavobacterium | 0.311687891 |
| ASV_84 | Firmicutes | Bacilli | Bacillales | Bacillaceae | Bacillus | 0.067380646 |
| ASV_85 | Proteobacteria | Gammaproteobacteria | Diplorickettsiales | Diplorickettsiaceae | Diplorickettsia | 0.018389518 |
| ASV_86 | Proteobacteria | Alphaproteobacteria | Rhizobiales | Rhizobiaceae | Aureimonas | 0.029484678 |
| ASV_88 | Proteobacteria | Alphaproteobacteria | Sphingomonadales | Sphingomonadaceae | Sphingomonas | 0.070869723 |
| ASV_89 | Proteobacteria | Gammaproteobacteria | Pseudomonadales | Pseudomonadaceae | Pseudomonas | 0.011844444 |
| ASV_90 | Proteobacteria | Alphaproteobacteria | Rhizobiales | Xanthobacteraceae | unclassified_Xanthobacteraceae | 0.525600139 |
| ASV_91 | Proteobacteria | Gammaproteobacteria | Burkholderiales | Comamonadaceae | unclassified_Comamonadaceae | 0.023800000 |
| ASV_92 | Proteobacteria | Alphaproteobacteria | Acetobacterales | Acetobacteraceae | unclassified_Acetobacteraceae | 0.060810141 |
| ASV_93 | Proteobacteria | Gammaproteobacteria | unclassified_Gammaproteobacteria | NA | NA | 0.020572431 |
| ASV_94 | Proteobacteria | Gammaproteobacteria | Burkholderiales | unclassified_Burkholderiales | NA | 0.055786581 |
| ASV_95 | Bacteroidota | Bacteroidia | Flavobacteriales | Blattabacteriaceae | Candidatus Karelsulcia | 0.051347176 |
| ASV_96 | Proteobacteria | Alphaproteobacteria | Rhizobiales | Rhizobiaceae | Allorhizobium-Neorhizobium-Pararhizobium-Rhizobium | 0.190088160 |
| ASV_99 | Proteobacteria | Alphaproteobacteria | Rhizobiales | Rhizobiales Incertae Sedis | Phreatobacter | 0.231697696 |
| ASV_100 | Actinobacteriota | Actinobacteria | Corynebacteriales | Nocardiaceae | Williamsia | 0.046217050 |
| ASV_108 | Bacteroidota | Bacteroidia | Chitinophagales | Chitinophagaceae | Asinibacterium | 0.007000000 |
| ASV_110 | Firmicutes | Bacilli | Lactobacillales | Streptococcaceae | Streptococcus | 0.015688062 |
| ASV_116 | Proteobacteria | Alphaproteobacteria | Sphingomonadales | Sphingomonadaceae | Altererythrobacter | 0.013695238 |
| ASV_118 | Proteobacteria | Gammaproteobacteria | Burkholderiales | Oxalobacteraceae | Candidatus Nasuia | 0.015255135 |
| ASV_119 | Bacteroidota | Bacteroidia | Flavobacteriales | Blattabacteriaceae | Candidatus Karelsulcia | 0.031817027 |
| ASV_125 | Firmicutes | Clostridia | NA | Hungateiclostridiaceae | Ruminiclostridium | 0.017555556 |
| ASV_132 | Proteobacteria | Gammaproteobacteria | Diplorickettsiales | Diplorickettsiaceae | Diplorickettsia | 0.015734465 |

*Note: Bold and italic are indicating the top three taxa (ASVs) with the highest score (mean decrease in accuracy/Gini)*

| Table S5. Endosymbiont bacteria associated with Cicadellidae species (hosts) | | | | |
| --- | --- | --- | --- | --- |
| Leafhoppers | | | ***n*** | **Symbionts** |
| Subfamily | **Tribe** | **Species** |  |  |
| Cicadellinae | Cicadellini | *Cofana spectra* | 8 | *Karelsulcia*; Sodalis^(5)^; Wolbachia^(1)^; Rickettsia^(5)^ Ca.* Lariskella*^(2)^* |
| Iassinae | Batracomorphini | *Batracomorphus angustatus* | 2 | *Karelsulcia*; Diplorickettsia** |
| Deltocephalinae | Chiasmini | *Exitianus indicus* | 11 | *Karelsulcia*; Nasuia^(5)^*  *Arsenophonus ^(2)^ Wolbachia^(11)^* |
|  |  | *Nephotettix nigropictus* | 5 | *Karelsulcia*; Nasuia**  *Diplorickettsia ^(1)^* |
|  |  | *Nephotettix virescens* | 34 | *Karelsulcia*; Nasuia* Arsenophonus^(1)^; Wolbachia^(2)^, Diplorickettsia ^(31)^* |
|  | Deltocephalini | *Maiestas dorsalis* | 12 | *Karelsulcia** |
|  |  | *Maiestas* sp. | 2 | *Karelsulcia** |
|  |  | Deltocephalini sp. | 4 | *Karelsulcia* & Nasuia** |
|  | Goniagnathini | *Goniagnathus punctifer* | 7 | *Karelsulcia** |
|  | Hecalini | *Hecalus arcuatus* | 4 | *Karelsulcia*; Sodalis^(2)^*  *Ca.* Lariskella*^(2)^* |
|  |  | *Hecalus* sp. | 4 | *Karelsulcia*; Wolbachia^(3)^* |
|  | Macrostelini | *Balclutha* sp. | 4 | *Karelsulcia*; Wolbachia** |
|  | Opsiini | *Hishimonus* sp. | 2 | *Karelsulcia** |
|  | Penthimiini | *Neodartus* sp. | 8 | *Karelsulcia** |
|  | Stenometopiini | *Stirellus capitatus* | 7 | *Karelsulcia** |
|  |  | *Stirellus* sp1. | 4 | *Karelsulcia** |
|  |  | *Stirellus* sp2. | 8 | *Karelsulcia*; Wolbachia^(5)^* |
| * Endosymbiont detected in every single specimen of the host species  ^(n)^ Number indicates proportion of specimens (out of n) in which the symbiont was detected, based on presence/absence | | | | |
