# Supplementary material for: Symbiont Diversity of Rice-Associated Leafhoppers (Cicadellidae) in the Tropical Floodplains of the Tonle Sap Lake, Cambodia
Source: Microb Ecol. 2025 Oct 17;88:109. doi: 10.1007/s00248-025-02619-9 (PMC12534257; doi:10.1007/s00248-025-02619-9)
Supplement: Supplementary file 2 [file 248_2025_2619_MOESM3_ESM.docx]

***Symbiont diversity of rice-associated leafhoppers (Cicadellidae) in the tropical floodplains of Tonle Sap Lake, Cambodia***

**Sophany Phauk^1,2^*, Lorenzo Assentato^2^, Sopha Sin^3^, Onnorong Uk^1^, Sophorn Hap^1^ and Olle Terenius^2^**

^1^	Department of Biology, Faculty of Science, Royal University of Phnom Penh, Cambodia

^2^	Department of Cell and Molecular Biology, Microbiology, Uppsala University, Uppsala, Sweden

^3^	Centre for Biodiversity Conservation, Faculty of Science, Royal University of Phnom Penh, Cambodia

*	Corresponding author

Email address: [sophany.phauk@icm.uu.se](mailto:sophany.phauk@icm.uu.se)

**Figure S1.** Phylogenetic tree of 17 Cicadellidae hosts was classified into 3 subfamilies (Iassinae, Cicadellinae and Deltocephalinae). Maximum Likelihood (ML) method with 1,000 bootstraps value of 32 cicadellid samples used in the analysis. An average of 658 bp portion of Mitochondrial Cytochrome Oxidase (COI) DNA barcode gene region. The analysis was made in the Molecular Evolutionary Genetics Analysis (MEGA 11 vrs.).

**Figure S2.** Positive Control: two samples from the ZymoBIOMICS Microbial Community Standard (Zymo Research) were used as positive controls to evaluate our protocol. When processed along the same pipeline together with the samples from this study, 6 out of 8 bacterial strains were identified correctly at the genus level. Salmonella enterica was classified only down to the Family level as Enterobacteriaceae, while Bacillus subtilis was undetected. Most of the bacteria had proportions close to the expected ones (14%), with Listeria being the main under-represented, and Enterococcus being slightly over-represented. This might indicate a bias in the community composition skewed towards Enterococcacea

**Figure S3.** Composition of symbiotic bacterial associated with Cicadellidae hosts at the genus level. (A) Bacterial composition in male and female *Nephotettix virescens* (green leafhopper). (B) Comparison of bacterial composition across eight cicadellid species from two different locations (provinces).

**Figure S4.** Alpha diversity of bacterial communities associated with *Nephotettix virescens* across different locations (A) and seasons (B) based on *Obs* richness and *Shannon* index. Figure (C) highlights key microbial taxa (ASVs) that most strongly contribute to differences in the bacterial community profiles between male and female *N. virescens*, as identified by Random Forest (RF) analysis. The plot shows the top tree taxa with the highest score (mean decrease in accuracy/Gini), indicating their importance in classification (Table S4). The abbreviations on the *x-axis* in panel A correspond to the following location: Battambang (Btb), Kampong Chhnang (Kpc), Kampong Thom (Kpt), Pursat (Pst) and Siem Reap (Sir) provinces.
